# Retinal Vibrations in Bacteriorhodopsin are Mechanically Harmonic but Electrically Anharmonic: Evidence From Overtone and Combination Bands

**DOI:** 10.3389/fmolb.2021.749261

**Published:** 2021-12-17

**Authors:** Victor A. Lorenz-Fonfria, Kiyoshi Yagi, Shota Ito, Hideki Kandori

**Affiliations:** ^1^ Institute of Molecular Science, Universitat de València, Paterna, Spain; ^2^ Theoretical Molecular Science Laboratory, RIKEN Cluster for Pioneering Research, Saitama, Japan; ^3^ Department of Life Science and Applied Chemistry, Nagoya Institute of Technology, Nagoya, Japan; ^4^ OptoBioTechnology Research Center, Nagoya Institute of Technology, Nagoya, Japan

**Keywords:** retinal, bacteriorhodopsin, anharmonicity, combinations and overtones, FTIR—spectroscopy, anharmonic vibrational calculations, vibrational quasi-degenerate perturbation theory, microbial rhodopsins

## Abstract

Fundamental vibrations of the chromophore in the membrane protein bacteriorhodopsin (BR), a protonated Schiff base retinal, have been studied for decades, both by resonance Raman and by infrared (IR) difference spectroscopy. Such studies started comparing vibrational changes between the initial BR state (all-*trans* retinal) and the K intermediate (13-*cis* retinal), being later extended to the rest of intermediates. They contributed to our understanding of the proton-pumping mechanism of BR by exploiting the sensitivity of fundamental vibrational transitions of the retinal to its conformation. Here, we report on new bands in the 2,500 to 1,800 cm^−1^ region of the K-BR difference FT-IR spectrum. We show that the bands between 2,500 and 2,300 cm^−1^ originate from overtone and combination transitions from C-C stretches of the retinal. We assigned bands below 2,300 cm^−1^ to the combination of retinal C-C stretches with methyl rocks and with hydrogen-out-of-plane vibrations. Remarkably, experimental C-C overtone bands appeared at roughly twice the wavenumber of their fundamentals, with anharmonic mechanical constants ≤3.5 cm^−1^, and in some cases of ∼1 cm^−1^. Comparison of combination and fundamental bands indicates that most of the mechanical coupling constants are also very small. Despite the mechanical quasi-harmonicity of the C-C stretches, the area of their overtone bands was only ∼50 to ∼100 times smaller than of their fundamental bands. We concluded that electrical anharmonicity, the second mechanism giving intensity to overtone bands, must be particularly high for the retinal C-C stretches. We corroborated the assignments of negative bands in the K-BR difference FT-IR spectrum by ab initio anharmonic vibrational calculations of all-trans retinal in BR using a quantum-mechanics/molecular mechanics approach, reproducing reasonably well the small experimental anharmonic and coupling mechanical constants. Yet, and in spite accounting for both mechanical and electrical anharmonicities, the intensity of overtone C-C transitions was underestimated by a factor of 4–20, indicating room for improvement in state-of-the-art anharmonic vibrational calculations. The relatively intense overtone and combination bands of the retinal might open the possibility to detect retinal conformational changes too subtle to significantly affect fundamental transitions but leaving a footprint in overtone and combination transitions.

## Introduction

The vibrations of atoms within molecules are deeply connected with the geometry and force of their chemical bonds. The reason is that the shape of the multidimensional potential energy surface holding the atoms of molecules ends defining the energy levels and the associated wavefunctions of the vibrational modes and, thus, their interaction with light. Therefore, it is not surprising that infrared spectroscopy, being based on the interaction of the electromagnetic field with quantized vibrational levels of molecules, holds a prominent position in the structural characterization of molecules. The applications of IR spectroscopy goes from classical studies determining bond distances of diatomic molecules in the gas phase (Struve, 1989), to modern applications on biological macromolecules (Koziol et al., 2015; Ghosh et al., 2017). Particularly successful has been the application of IR difference spectroscopy to light-sensitive proteins, where spectral changes following light excitation are selectively resolved, often as a function of time (Kötting and Gerwert, 2013; Kottke et al., 2017). Nevertheless, the interpretation of changes in positions, intensities and/or widths of bands in IR difference spectra of proteins is often based on simple rules of thumb about the effects of H-bonding, polarity and vibrational coupling on vibrational properties (Barth and Zscherp, 2002; Lorenz-Fonfria, 2020), rarely specific enough to provide quantitative atomistic predictions. In this context, vibrational calculations are gaining popularity as a tool to guide the interpretation of experimental spectra in atomic terms, resolving ambiguities about protonation states and H-bonds of groups (Domratcheva et al., 2016; Nakamura and Noguchi, 2016, 2017; Peuker et al., 2016), or tautomers (Domratcheva et al., 2016), complementing ambiguous or missing information in X-ray crystallographic structures of proteins. However, the utility of vibrational computations to interpret experimental IR spectra from biomolecules is still limited by our ability to accurately calculate IR spectra from input molecular structures.

One common approximation in calculations of vibrational frequencies is the assumption that atomic displacements from the energy minimum in a vibrational mode are small and, thus, that the potential energy surface can be expanded along selected molecular coordinates (often the normal mode coordinates) discarding higher than quadratic terms. This approximation, known as mechanical harmonicity, leads to harmonic wavefunctions, the quantum equivalent to the classical harmonic oscillator. In the quantum harmonic oscillator, the energy gap between consecutive vibrational states is constant, and usually expressed as the frequency of a photon: the harmonic frequency. This frequency is generally expressed in wavenumbers, using units of cm^−1^. In addition, the calculation of the IR intensities, related to the probability of a transition between two vibrational states by light absorption, often assumes that the dipole moment of the molecule changes linearly along the selected coordinate, an approximation known as electrical harmonicity. These two approximations, known as the harmonic oscillator linear dipole approximation, leads to the familiar quantum harmonic oscillator (Struve, 1989), with two main characteristics. 1) The vibrational energy levels are equally spaced, with a constant energy gap between consecutive vibrational states: ground state (*n* = 0), first excited state (*n* = 1), second excited state (*n* = 2), etc. 2) Only fundamental transitions (*n* = 0 → *n* = 1) and hot transitions (*n* = *i* → *n* = *i* ± 1, with *i* > 0), the latter rare at room temperature, are allowed by selection rules.

An experimental hallmark for the breakdown of the harmonic approximation is the observation of overtone bands, i.e., transitions from the vibrational ground state to the second and further excited states (*n* = 0 → *i*, with *i* = 2, 3). Another hallmark are combination bands, i.e., light absorption simultaneously driving two vibrations (*n* and *m*) from their vibrational ground state to the first [(*n* = 0, *m* = 0) → (*n* = 1, *m* = 1)] or further excited states [(*n* = 0, *m* = 0) → (*n* = 1, *m* = 2); (*n* = 0, *m* = 0) → (*n* = 2, *m* = 1); (*n* = 0, *m* = 0) → (*n* = 2, *m* = 2), etc.].

Mechanical anharmonicity can be easily diagnosed and quantified because it affects the energy (frequency) difference between fundamental and overtone transitions, as a result of the energy levels being no longer equally spaced. For instance, the mechanical anharmonic constant of mode *i*, *X*_*ii*_, can be determined as the fundamental minus half the first overtone frequency (Sandorfy et al., 2006). Mechanical anharmonicities have been most studied for X-H stretching vibrations (X = C, N, O, S), typically ranging from 50 to 85 cm^−1^ when free from H-bonding (Struve, 1989). Data for other vibrations is scarcer. For X-Y stretches (X and Y = C, N or O) of diatomic molecules (Struve, 1989) or for C=O stretches from carbonyls (Groh, 1988), mechanical anharmonic constants are considerably smaller, between 12 and 14 cm^−1^, and for C-X stretches (X = F or Cl), as small as 5 cm^−1^ (Groh, 1988). On the other hand, the mechanical coupling constant between the *i* and *j* modes, *X*_*ij*_, is the sum of the *i* and *j* fundamentals minus the frequency of their first combination band (Sandorfy et al., 2006). As a reference, the coupling constant between symmetric and asymmetric X-H vibrations (X = N or O) is around 90–160 cm^−1^ (Sandorfy et al., 2006).

Mechanical anharmonicity not only modifies the energy of vibrational levels and, thus, the frequency of fundamental and overtone/combination transitions, but it also modifies the wavefunction of the vibrational states. Consequently, it gives IR intensity (absorption probability) to overtone and combination transitions, forbidden (i.e., with null IR intensity) under the harmonic approximation. But, while the presence of overtone and combination bands is often considered a sign of mechanical anharmonicity, electrical anharmonicity alone is sufficient to give IR intensity to overtones and combination bands, even for purely harmonic wavefunctions, affecting even the intensity of fundamental transitions (Sandorfy et al., 2006; Panek and Jacob, 2016). Because the IR intensity of overtone and combination bands is simultaneously affected by mechanical and electrical anharmonicities, in practice both contributions are experimentally difficult to disentangle from each other. Consequently, the role played by electrical anharmonicities on shaping IR spectra in biological macromolecules is yet to be demonstrated experimentally to our knowledge.

**FIGURE 1:**
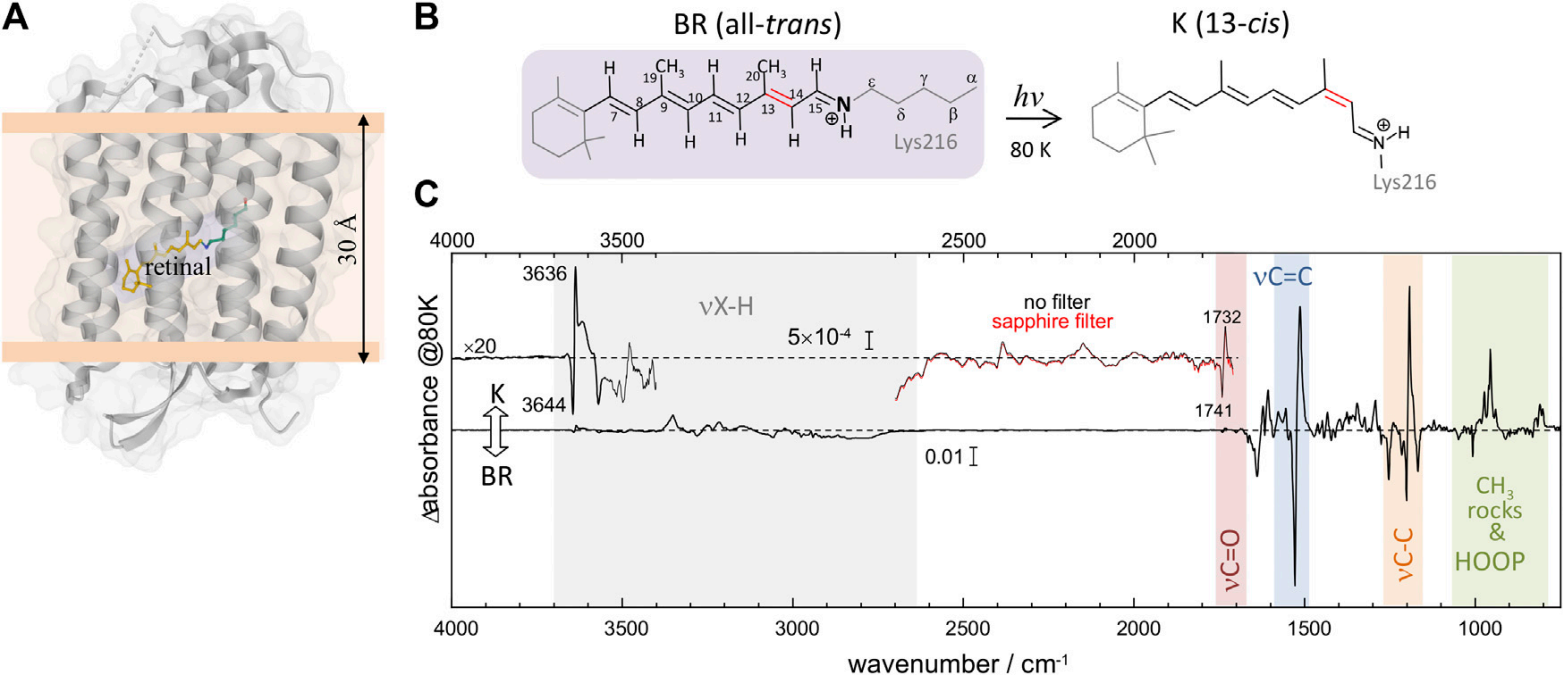
Structural and vibrational differences between bacteriorhodopsin (BR) in its initial state (all-*trans* retinal) and in the K intermediate (13-*cis* retinal). **(A)** Structure of BR in the initial state (pdb 1C3W) (Luecke et al., 1999), highlighting the location of the retinal chromophore (in yellow), forming a protonated Schiff base (PSB) with Lys216. **(B)** Schematic representation of the PSB retinal chromophore in the initial state of BR (all-*trans*, 15-anti conformation) and in the K intermediate (13-*cis*, 15-anti conformation), including the numbering of relevant carbon atoms. **(C)** K-minus-BR difference FT-IR spectrum obtained by illumination at 80 K, with highlighted regions corresponding to X-H stretches, carboxylic C=O stretches, retinal C=C and C-C stretches, retinal CH_3_ rocks, and retinal hydrogen out of plane (HOOP) ethyl wags. The inset above 3,400 cm^−1^, besides illustrating the noise level, shows bands at 3,644 (–) and 3,636 (+) cm^−1^ from the νO-H of internal dangling water 401. The inset between 2,600 and 1,710 cm^−1^ shows bands at 1732 (+) and 1741 (–) cm^−1^ from carboxylic νC = O vibrations of Asp115, as well as several bands never reported before. Measurements with a sapphire window, acting as a high pass filter (cutoff at ∼1,650 cm^−1^), exclude that double-modulation artifacts might contribute to the absorption changes between 2,600 and 1,710 cm^−1^.

Bacteriorhodopsin (BR) is a light-driven proton-pump membrane protein that belongs to the family of microbial rhodopsins, a family present in the genome of cells from all kingdoms of life (Ernst et al., 2014). It contains seven transmembrane helices and a protonated Schiff base (PSB) retinal chromophore (Figure 1A). Photoisomerization of the PSB retinal from all-trans to 13-*cis* conformation (Figure 1B) starts a photocyclic reaction involving orchestrated changes of the conformation and protonation and state of the retinal, as well as of key residues, with the final result of a proton being vectorially transported from the cytoplasm to the extracellular side (Ernst et al., 2014). The photocycle of BR involves various intermediate states which can be trapped at low temperatures (Balashov and Ebrey, 2007). The intermediate K, the first thermally metastable state of the photocycle, can be trapped by illumination at 80 K (Rothschild and Marrero, 1982; Balashov and Ebrey, 2007).

We report on previously unnoticed bands between 2,550 and 2,300 cm^−1^ in the FT-IR difference spectrum between the K intermediate and the initial BR state at 80 K (K-BR for short). Based on their wavenumber, we assigned most of them to overtone and combination bands of C-C stretching vibrations of the retinal: the negative bands to the initial state of BR (all-trans PSB retinal) and the positive bands to the K intermediate (13-*cis* PSB retinal). Comparing bands from fundamental C-C stretches with their putative overtones, we concluded that retinal C-C stretching vibrations are virtually mechanically harmonic both in the initial state of BR and in the K intermediate, with anharmonic mechanical constants between +1.0 cm^−1^ and +3.5 cm^−1^. The mechanical quasi-harmonicity for the retinal C-C stretches reported here sharply contrasts with the relative area of the overtone bands, only ∼50–100 times smaller than for their fundamentals. We take this observation as indication that retinal vibrations in bacteriorhodopsin are notably electrically anharmonic.

We complemented the experimental observations with two state-of-the-art ab initio anharmonic vibrational calculations for all-trans PSB retinal in the BR initial state. Calculated spectra correctly reproduced experimental bands from fundamental C-C vibrations, as well as many putative overtones/combination bands, helping in their assignment. The calculations also reproduced the small mechanical anharmonic and coupling constants of the C-C stretches. However, they underestimated by a factor of 4–20 the relative intensity of the overtone bands with respect to their fundamentals. This discrepancy might indicate potential limitations in current state-of-the-art anharmonic calculations, for instance when electrical anharmonicities are dominant, as in the present case.

Overall, our results provide a solid example for the role that electrical anharmonicity plays in determining the intensity of overtone and combination bands. We believe that our experimental results, rich in details, could be useful to benchmark future developments in anharmonic vibrational calculations. In addition, the present detection and assignment of overtone and combination bands from the retinal, can be the starting point for future works exploiting these overtone and combination bands to gather information about retinal conformational changes that might be too subtle to affect fundamental bands.

## Materials and Methods

### FT-IR Difference Spectroscopy

Purple membranes were isolated from *Halobacterium salinarum* as described (Oesterhelt and Stoeckenius, 1974). Around 25 μl of a 6 mg/ml solution of BR in purple membranes (3 mM MES, 2 mM NaCl, pH 6.5) was dried at ambient humidity on top of a BaF_2_ window of 18 mm diameter. The resulting film was rehydrated through the vapor phase provided by 4 μl of a mixture of water/glycerol (9/1 w/w) distributed in 3–5 drops placed nearby the film and closed with a second BaF_2_ window using a 0.5 mm thick silicone spacer. The sample was placed on a cryostat (OptistatDN, Oxford) coupled to a FT-IR spectrometer (Cary 670, Agilent) equipped with a MCT detector and purged with dry nitrogen. The BaF_2_ windows were placed roughly perpendicular to the IR beam but slightly tilted. After letting 30–45 min to complete the hydration process, the sample was adapted to light by 2 min of illumination (>530 nm, IR filtered), and cooled down to 80 K. The FT-IR absorption spectrum of the hydrated film of BR at 80 K is shown in Supplementary Figure S1. The light-induced K-BR FT-IR difference spectrum was obtained by 1 min of illumination using 540 ± 10 nm light. The back-conversion from K to the BR dark-state was done with >670 nm illumination for 1 min, to obtain the BR-K spectrum. Interferograms at 2 cm^−1^ resolution were collected before and after the illumination to obtain an FT-IR difference spectrum with a total of 128 accumulated scans. Alternating illumination to promote the forward and backward photoconversion was repeated four times, resulting in averaged K-BR and BR-K with 512 coadded scans each (Supplementary Figure S2). Further averaging the K-BR and the minus BR-K difference spectra cancelled trace absorption contributions from water vapor and CO_2_ (Supplementary Figure S2). In spite the high absorbance of the BR film used (Supplementary Figure S1), the obtained K-BR IR difference spectrum was almost indistinguishable from that obtained with a sample with three times less absorbance (Supplementary Figure S3), except at regions with a background absorbance higher than 2. Thus, when displaying the whole K-BR difference spectrum (Figure 1C), absorbance changes in regions with a background absorbance above 2 were taken from a sample with three times less protein (after an appropriate rescaling). K-BR difference spectra were measured with and without a sapphire window placed in the optical path, which fully blocks light below 1,600 cm^−1^, without any appreciable discrepancy in the 2,600–1,800 cm^−1^ region between them. When using an older FT-IR spectrometer (Biorad FTS-40), used in a previous publication (Ito et al., 2018), the use of a sapphire window was required to disentangle true overtone and combination band signals from double modulation artifacts (Supplementary Figure S4).

### Anharmonic Vibrational Calculations

The atomistic model of BR was constructed based on an X-ray crystal structure with PDB ID 1C3W (Luecke et al., 1999). Missing residues, between 157–161, were complemented using MODELLER 9.14. (Šali, 2014). The protonation state of titratable residues was determined based on pKa values obtained by PROPKA 3.1. (Olsson et al., 2011): Asp96, Asp115, and Glu194 were protonated, while other residues were kept ionized. Hydrogen atoms were added using the HBUILD utility of CHARMM (Brooks et al., 2009), and relaxed by performing molecular dynamics (MD) simulations for 100 ps with the positions of the heavy atoms kept fixed.

The last structure of the MD trajectory was used for the subsequent QM/MM calculations (Yagi et al., 2019). In the QM/MM calculation, the retinal, as well as three nearby residues (sidechains of Asp85 and Asp212 beyond C_β_ and of Lys216 beyond C_γ_) and a crystal water molecule (Wat402), were included in the QM region (74 atoms in total), treated at the DFT level. For the MM region, which includes the remaining protein and water molecules, we used CHARMM36 (Best et al., 2012) and TIP3P (Jorgensen et al., 1983) force fields, respectively. The energy minimization was followed by harmonic vibrational analysis, with a partial Hessian which includes all atoms from the retinal and from Lys216 beyond C_δ_ (56 atoms, 168 vibrational modes).

Anharmonic vibrational calculations were carried out following three schemes. In the first scheme (Scheme A), the vibrational calculation was performed in 116 dimensions neglecting 52 low frequency modes (<800 cm^−1^). Optimized-coordinate vibrational self-consistent field (oc-VSCF) was first carried out with a cubic force field to optimize the vibrational coordinates (Yagi et al., 2012; Yagi and Otaki, 2014). These coordinates were used to generate the anharmonic potential energy surface (PES). The quartic force field (Yagi et al., 2004) was generated in 116 dimensions. The grid potential (Yagi et al., 2000) was generated in one-dimension for 83 modes with 11 grid points, including C-H and N-H stretching modes, and in two-dimension with 9 grid points for the combination of modes 72/X, 78/X, and X/X, where X represents modes 86, 89, 92, or 93. Note that the dipole moment surfaces (DMS) were obtained simultaneously with the PES. Then, VSCF and the second-order vibrational quasi-degenerate perturbation theory (VQDPT2) calculations (Yagi et al., 2008; Yagi and Otaki, 2014) were performed to obtain an IR line spectrum (transition intensities vs. transitions energies). VSCF was carried out with 11 harmonic oscillator basis functions for each mode, and VQDPT2 was carried out setting the maximum sum of excitation to 4 and the number of P space generation to 1. In the second scheme (Scheme B), the calculation was performed in only 8 selected modes consisting of four C-C stretching modes (modes 86, 89, 92, and 93), and modes 70, 72, 82, 136. The PES and DMS were constructed using a grid method with 11 and 9 grid points for the one-mode and the two-mode part, respectively (Yagi et al., 2000). Finally, the IR line spectrum was obtained by VSCF and vibrational configuration interaction (VCI) calculations using the resulting PES and DMS. VSCF was performed with 11 harmonic oscillator basis functions on each mode, and VCI was carried out at the level of three-mode excitation, setting the maximum sum of quantum number to 6. Supplementary Table S2 includes the main VSCF configuration functions contributing to calculated fundamental and excited (overtone/combination) states involving retinal C-C stretching vibrations, both for Scheme A and Scheme B.

To highlight the effect of electrical anharmonicity on the C-C overtone/combination bands, we performed a third type of calculation (Scheme C). In this vibrational calculation, we reused the same 8 coordinates and DMS calculated for Scheme B, but we applied a harmonic PES. The purpose of Scheme C was to disentangle the contribution of mechanical and electrical anharmonicities to the calculated intensities of overtone and combination bands in Scheme B.

The QM/MM calculations were performed using a development version of GENESIS 1.6.1. (www.r-ccs.riken.jp/labs/cbrt) (Jung et al., 2015; Kobayashi et al., 2017). The QM calculation was carried out at the level of B3LYP-D3 (Lee et al., 1988; Becke, 1993; Grimme et al., 2010) with cc-pVDZ (Scheme A) or cc-pVTZ (Scheme B) basis sets (Dunning, 1989) using Gaussian16 (http://gaussian.com/gaussian16/) (Frisch et al., 2016) or Q-Simulate (see https://qsimulate.com/), as done previously (Yagi et al., 2021). The vibrational calculation was performed using SINDO 4.0 (available at tms.riken.jp/en/research/software/sindo).

### Data Postprocessing

IR line spectra, obtained from anharmonic vibrational calculations, were convoluted with a Lorentzian band to facilitate their comparison with the experimental spectra. For fundamental transitions, we used a Lorentzian full width at half height (FWHH) of 7 cm^−1^, and for overtone and combination transitions a FWHH of 14 cm^−1^. This choice reflects the experimental observation that overtone bands appear twice broader than fundamental bands. On the other hand, to improve the resolution of overlapping bands, the experimental K-BR difference FT-IR spectrum was band-narrowed by Fourier self-deconvolution (Kauppinen et al., 1981) as implemented in the Matlab App FourierDataProcessing, available at https://www.mathworks.com/matlabcentral/fileexchange/92573-fourierdataprocessing.

## Results and Discussion

### K-Minus-BR FT-IR Difference Spectrum

**FIGURE 2:**
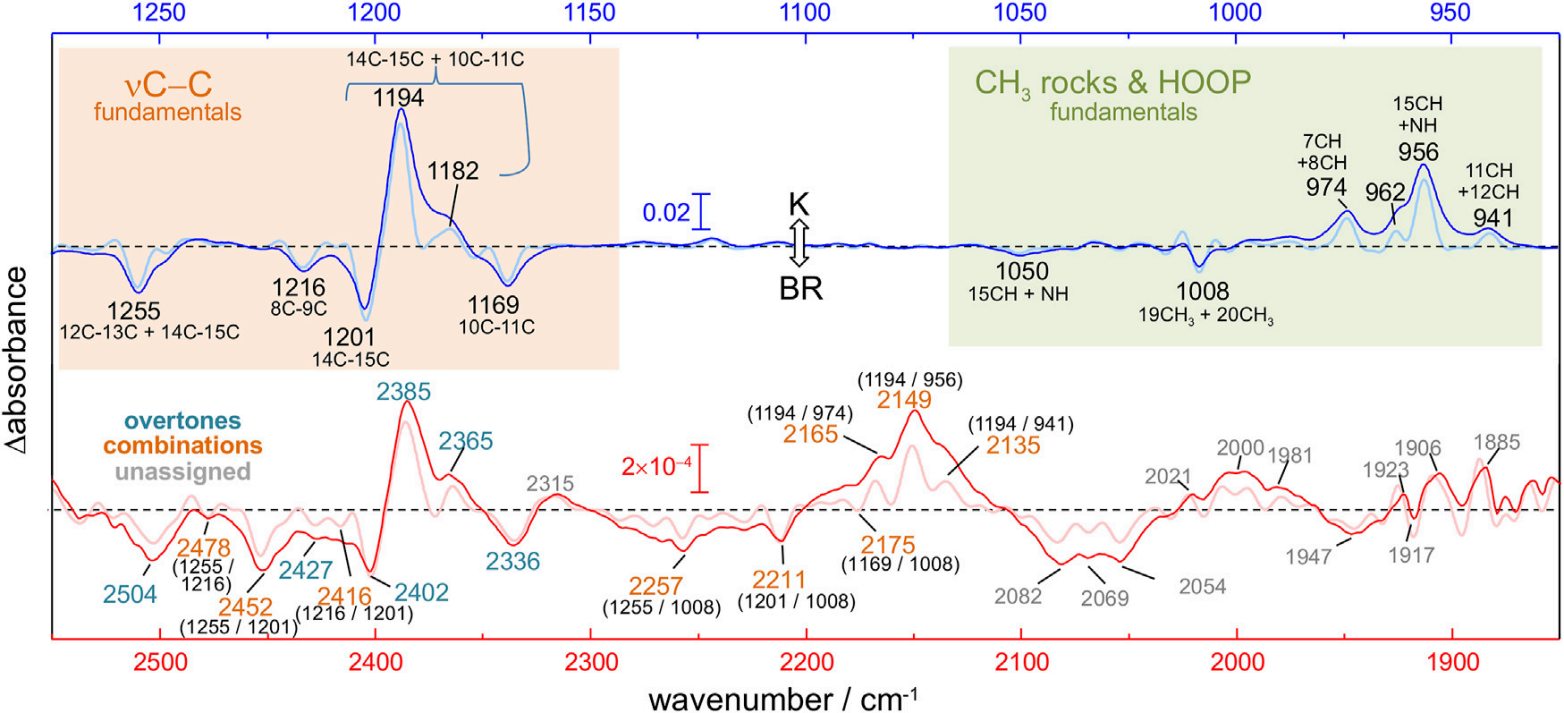
K-BR difference FT-IR spectrum at 80 K. Positive bands come from vibrations in the K intermediate and negative bands in the initial BR state. The figure compares fundamental transitions from retinal C-C stretches, CH_3_ rocks and HOOP vibrations in the 1,275–925 cm^−1^ region (blue trace), with potential overtones and combinations in the 2,550–1,850 cm^−1^ region (red trace). The K-BR spectrum is also displayed after band narrowing with Fourier self-deconvolution (pale blue and pale red traces). For combination bands, the wavenumber of the two putative fundamental frequencies is given in brackets.

Light-induced FT-IR difference spectra of BR at low temperature have been amply measured and characterized in the past (Bagley et al., 1982; Rothschild and Marrero, 1982; Siebert and Mäntele, 1983; Earnest et al., 1986; Fischer et al., 1994; Kandori et al., 1998), as reviewed (Maeda, 1995; Kandori, 2020). Illumination at 80 K with 540 nm traps the K intermediate (Rothschild and Marrero, 1982; Balashov and Ebrey, 2007), the first metastable intermediate after all-*trans* to 13-*cis* photoisomerization of the PSB retinal. The trapped K intermediate reverts to the BR state with >670 nm illumination (Rothschild and Marrero, 1982; Balashov and Ebrey, 2007). Figure 1C shows the K-minus-BR FT-IR difference spectrum (K-BR spectrum for short), where positive bands correspond to the K intermediate (13-*cis* PSB retinal) and negative bands correspond to the initial BR state (all-*trans* PSB retinal), BR state for short. In the K intermediate most of the structural changes are restricted to the chromophore and its vicinity (Neutze et al., 2002; Wickstrand et al., 2019), and, thus, most of the resolved bands in the K-BR spectrum come from vibrational modes located at the retinal molecule (Rothschild et al., 1984). At ∼1,600–1,500 cm^−1^ and at ∼1,275–1,150 cm^−1^ we find bands from fundamental transitions of retinal C=C and C-C stretches, and between 1,050 and 750 cm^−1^ fundamental transitions of methyl rocks and hydrogen out of plane (HOOP) vibrations (Smith et al., 1987; Maeda, 1995). The specific assignment of bands between 1,275 and 900 cm^−1^ is given in Figure 2 (blue trace), and assignments for the negative bands are collected in Table 1. Besides retinal vibrations, we have bands at 1,741 (–) and 1,732 (+) cm^−1^ from fundamental transitions of the carboxylic C=O stretching of Asp115 (Braiman et al., 1988), reflecting its stronger H-bonding with Thr90 in the K intermediate (Welke et al., 2013). Between ∼3,700 and ∼2,700 cm^−1^ we have fundamental transitions from X-H vibrations (X = O, N, and C), including bands at 3,644 (–) and 3,636 (+) cm^−1^ from the O-H stretch of the dangling internal water 401 (Hayashi et al., 2004).

**TABLE 1:** Assignment of bands to fundamental vibrational transitions of the retinal in the BR state.

	Experiment		Scheme A^a^			Scheme B^b^		
Assignment	ν/cm^−1^	Area (relative)^c^	State	ν/cm^−1^ (Δν)^d^	Intensity (relative)^e^	State	ν/cm^−1^ (Δν)	Intensity (relative)
19C/20C rock	1,008	0.06 (0.20)	72_1_	1,002 (–6)	191 (0.49)	72_1_	1,034 (+26)	123 (0.36)
H15C = NH HOOP	1,050	0.09 (0.30)	82_1_	1,040 (–10)	73 (0.19)	82_1_	1,108 (+58)	64 (0.18)
10–11C	1,169	0.29 (1.0)	86_1_	1,199 (+30)	389 (1.0)	86_1_	1,211 (+42)	345 (1.0)
14–15C	1,201	0.59 (2.0)	89_1_	1,232 (+31)	888 (2.3)	89_1_	1,252 (+51)	261 (0.76)
8–9C	1,216	0.18 (0.62)	92_1_	1,225 (+9)	28 (0.07)	92_1_	1,264 (+48)	771 (2.2)
12–13C/14–15C	1,255	0.37 (1.2)	93_1_	1,269 (+14)	649 (1.7)	93_1_	1,300 (+45)	297 (0.86)

a VQDPT2@B3LYP-D3/cc-pVDZ.

b VCI@B3LYP-D3/cc-pVTZ.

c Experimental area in the K-BR spectrum, in cm^−1^ (in brackets relative to the 10–11C fundamental).

d Δν, variation between the experimental and calculated wavenumber.

e Calculated intensity in km/mol (in brackets relative to the intensity of the state 86_1_).

### Bands in the 2,550–1,800 cm^−1^ Spectral Range

In the K-BR difference spectrum we find previously unreported bands, extending from 2,550 to 1,800 cm^−1^ (Figure 1C, insert), in a region where bands from fundamental vibrational transitions rarely appear in proteins (Adhikary et al., 2017). These bands are reproduced in both in the K-BR and in the BR-K difference spectrum (Supplementary Figure S2) and display an intensity far above the noise level (Figure 1C, Insert). In addition, they are unaffected by placing in the optical path a sapphire window acting as a high pass optical filter (Figure 1C, insert, compare red and black traces), discarding that they could be ghost bands originated from double modulation artifacts in the interferometer (Ito et al., 2018). Although fundamental transitions from stretches of triple bonds (e.g., C≡C) and S-H groups can contribute to this spectral region, such chemical groups are absent in BR. We also expect contributions from fundamental transitions of X-D stretches in this spectral range. However, potential bands from X-D stretches are expected to be at least 100 times smaller than the bands observed here upon considering the natural abundance of deuterium (0.016%) and the intensity of X-H bands in the K-BR difference spectrum (Figure 1C).

Upon reasonably discarding that these new bands could originate from fundamental transitions, we explored their assignment to overtone and/or combination transitions. For that, we compared the spectral features between 2,550 and 1,800 cm^−1^ (Figure 2, red trace) with those between 1,275 and 900 cm^−1^ (Figure 2, blue trace). To further facilitate the spectral comparison of both regions, we mathematically narrowed the K-BR spectrum using Fourier self-deconvolution, FSD (Figure 2, pale blue and pale red traces). Laying both spectral regions on top of each reveals some remarkable similitudes between them (Figure 2). Several bands that align nicely with each other can be reasonably assigned, based on their wavenumber and appearance, to overtones of retinal C-C stretches (Figure 2, bottom, cyan labels). On a similar basis, several bands can be reasonably assigned to the combination of fundamentals, either between retinal C-C stretches, between retinal C-C stretches and methyl rocks, or between retinal C-C stretches and HOOP vibrations (Figure 2, bottom, orange labels). We confirmed that all the band discussed hereafter are, before and after FSD, fully reproducible (Supplementary Figure S6). The assignments of the negative bands (BR state) are collected in Table 2. These assignments will be later further justified in view of anharmonic spectral calculations. But before that, we will briefly review in the next section the assignment of bands in the 1,275 and 900 cm^−1^ region to fundamental transitions from vibrations of the PSB retinal.

**TABLE 2:** Assignment of bands to overtone and combination vibrational transitions of the retinal in the BR state.

	Experiment				Scheme A					Scheme B				
Assignment	ν/cm^−1^	*X* ^a^/cm^−1^	Area^d^	F/O^b^	State	ν/cm^−1^	*X* ^a^/cm^−1^	I^d^	F/O^b^	State	ν/cm^−1^	*X* ^a^/cm^−1^	I^d^	F/O^b^
19C/20C rock +10–11C	2,175	+1.7	0.0009	—	72_1_86_1_	2,201 (+26)^e^	−0.1	0.25	—	72_1_86_1_	2,248 (+73)^e^	−3.2	0.21	—
19C/20C rock +14–15C	2,211	−1.7	0.0036	—	72_1_89_1_	2,235 (+24)	+0.1	0.88	—	72_1_92_1_	2,302 (+91)	−3.8	1.29	—
19C/20C rock +12–13C/14–15C	2,257	+6.4	0.0020	—	72_1_93_1_	2,271 (+13)	+0.3	0.19	—	72_1_93_1_	2,337 (+80)	−2.8	0.17	—
(10–11C)_2_	2,336	+1.5	0.0059	50	86_2_	2,395 (+59)	+0.9	1.53	260	86_2_	2,422 (+86)	−0.2	1.43	240
10–11C + 14–15C	ND^c^	—	—	—	89_1_86_1_	2,430	+0.6	0.78	—	89_1_86_1_	2,466	−3.5	0.48	—
10–11C + 8–9C	ND	—	—	—	92_1_86_1_	2,423	+1.3	0.08	—	92_1_86_1_	2,478	−3.2	1.23	—
(14–15C)_2_	2,402	+1.0	0.0073	80	89_2_	2,456 (+54)	+3.9	0.50	1800	89_2_	2,503 (+101)	−0.1	0.79	330
8–9C + 14–15C	2,416	+2.7	0.0015	—	92_1_89_1_	2,462 (+56)	−5.1	1.11	—	92_1_89_1_	2,519 (+103)	−3.1	0.47	—
(8–9C)_2_	2,427	+2.8	0.0016	110	92_2_	2,450 (+23)	+0.6	0.08	340	92_2_	2,529 (+102)	−0.5	0.69	1700
14–15C + 12–13C/14–15C	2,452	+3.9	0.0068	—	93_1_89_1_	2,498 (+46)	+2.1	0.92	—	93_1_89_1_	2,556 (+104)	−4.4	0.52	—
8–9C +12–13C/14–15C	2,478	+6.1	0.0010	—	93_1_92_1_	—	+1.2	0.02	—	93_1_92_1_	2,567 (+89)	−3.3	0.70	—
(12–13C/14–15C)_2_	2,504	+3.5	0.0064	60	93_2_	2,535 (+31)	+1.1	0.67	970	93_2_	2,604 (+100)	−1.8	0.70	420

a *X* represents the mechanical anharmonic constant for overtones, and the coupling constant for combinations.

b Fundamental to overtone area ratio (for experimental spectra) and intensity ratio (for calculated spectra).

c ND: not detected.

d Band area in cm^−1^, and IR intensity in km/mol.

e Δν, variation between the experimental and calculated wavenumber.

### Fundamental Transitions From Vibrations of the PSB Retinal

**FIGURE 3:**
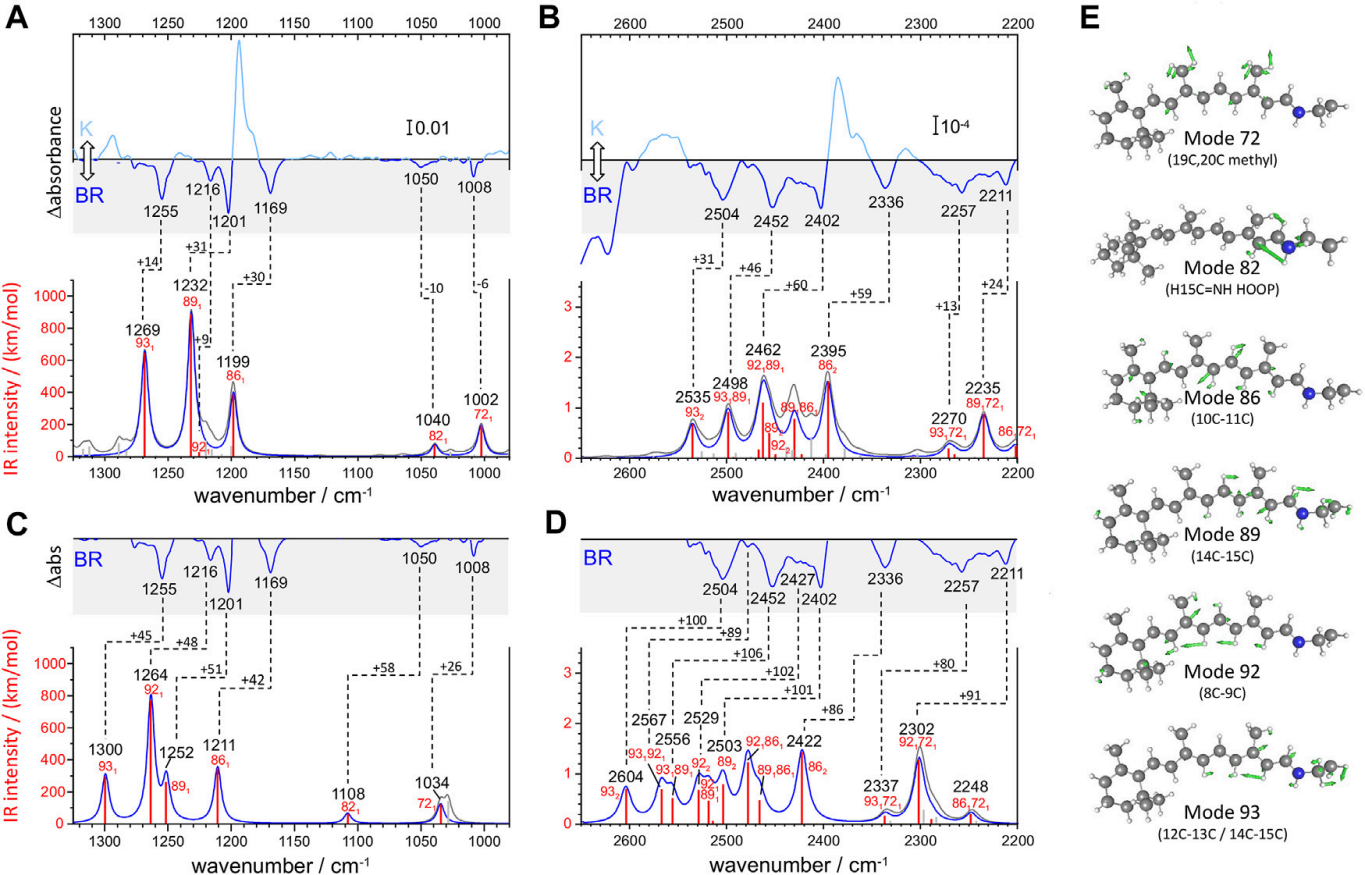
Calculated versus experimental IR spectra of all-*trans* PSB retinal in BR. **(A,B)** Calculation with scheme A vs. experimental K-BR spectrum. **(C,D)** Calculation with scheme B vs. experimental K-BR spectrum (only the negative bands from the BR state are shown). Bands assigned to a common vibrational transition are connected by dashes lines. See text and Materials and Methods for details about calculations with scheme A and scheme B. **(A–D)** Wavenumbers and IR intensities of calculated vibrational transitions (gray vertical lines). Transitions from modes with experimentally detected fundamentals (modes 72, 82, 86, 89, 92, and 93) are highlighted in red. Spectra were generated from the calculated intensity of vibrational transitions by convolution with a Lorentzian band (gray trace and blue traces), of either **(A,B)** 7 cm^−1^ or **(C,D)** 14 cm^−1^ full width at half height. Transitions are labelled as *x*_1_, *x*_2_, or *x*_1_ *y*_1_, for fundamental, overtones and combination transitions, respectively. **(E)** Atom displacements for some selected vibrational modes.

We start our assignment with the retinal C-C stretches. For the assignment of the negative bands in Figure 2 (blue spectrum), we relayed on previous works using BR reconstituted with isotopically labelled retinal (Gerwert and Siebert, 1986; Smith et al., 1987) and vibrational harmonic calculations of the PSB retinal in the BR state (Smith et al., 1987; Babitzki et al., 2009). The bands at 1,201 cm^−1^ and at 1,169 cm^−1^ correspond mostly to a 14–15C stretching and a 10–11C stretching vibration of the PSB retinal, respectively (Gerwert and Siebert, 1986; Smith et al., 1987). Incidentally, the numbering of the atoms of retinal atoms can consulted in Figure 1B. The negative band at 1,255 cm^−1^ has been assigned to coupled 12–13C and 14–15C stretches, with additional contributions from in-plane methyl rocks of 14C and 15C and to in-plane methylene vibrations of C_ε_ of Lys216 (Smith et al., 1987; Babitzki et al., 2009). Finally, the small negative band at 1,216 cm^−1^ corresponds mostly to a 8–9C stretch, with some 14–15C and 9–19C character (Smith et al., 1987). The negative band at 1,008 cm^−1^ (Figure 2, blue spectrum) has been assigned to a symmetric in-plane rocking combination of the 19C and 20C methyl groups of all-*trans* PSB retinal (Smith et al., 1987; Maeda et al., 1995). The negative band at 1,050 cm^−1^ (Figure 2, blue spectrum), remains unassigned to our best knowledge. Our anharmonic vibrational calculations, which we will describe below, assigns a similar band to an in-phase H15C = NH HOOP vibration (see mode 82 in Figure 3A and in Figure 3E). In agreement with this tentative assignment, this band vanishes upon deuteration of the NH group of PSB retinal by incubation of BR in D_2_O (Supplementary Figure S5).

For the assignment of positive bands, corresponding to 13-*cis* PSB retinal, we relayed exclusively on previous experimental work using BR reconstituted with isotope labelled retinal (Gerwert and Siebert, 1986; Fahmy et al., 1991; Weidlich and Siebert, 1993). Vibrational calculations for the retinal in the K intermediate are not available to our knowledge. The positive band at 1,194 cm^−1^ and the shoulder at 1,182 cm^−1^ (Figure 2, blue spectrum) originates from the mixing of the 14–15C and 10–11C stretches of 13-cis PSB retinal (Gerwert and Siebert, 1986). Regarding HOOP bands, at 974, 962, 956 and 941 cm^−1^ in Figure 2 (blue spectrum), only two of them, at 962 and 941 cm^−1^, are not affected upon deuteration of either N or 15C. Thus, the intense positive band at 956 cm^−1^ has been assigned to the H15C = NH HOOP mode (Maeda et al., 1991; Maeda, 1995). For the remaining bands tentative assignments have been proposed, but with rather weak experimental evidence. For instance, the positive band at the 941 cm^−1^ has been assigned to the H11C = 12CH HOOP (Earnest et al., 1986; Weidlich et al., 1997) and the positive band at 974 cm^−1^ to the H7C = 8CH HOOP mode (Fahmy et al., 1991).

### Anharmonic Vibrational Calculations of All-*trans* PSB Retinal

We conducted two anharmonic vibrational calculations of all-*trans* PSB retinal in the BR state, following two schemes: Scheme A (Figures 3A,B) and Scheme B (Figures 3C,D). The technical characteristics of these two anharmonic calculations are detailed in Materials and Methods. Briefly, our calculations started with a QM/MM energy minimization of the BR structure, with the QM region including the retinal, Lys216 from C_γ_, Asp85 and Asp212 from C_β_, and internal water molecule 401. This was followed by a harmonic vibrational calculation, restricted to the retinal and Lys216 (from C_γ_). From the resulting 158 vibrational modes, 6 of them had clear corresponding bands in the experimental K-BR spectrum (Figure 3). Mode 86 is mostly a 10‐11C stretching; mode 89 is mostly a 14-15C stretching; mode 92 is mostly a 8-9C stretching; and mode 93 as mostly a 12–13C/14–15C stretching (Figure 3E). In addition, we identified mode 72 as a 19C + 20C methyl rock and mode 82 as a H15C = NH HOOP (Figure 3E).

For the anharmonic vibrational calculations, we computed the potential energy surface (PES) and the dipole moment surface (DMS) for some selected normal mode coordinates. In Scheme A (Figures 3A,B), 116 coordinates with higher energy were set to be active. Anharmonic frequencies and intensities were calculated by vibrational self-consistent field (VSCF) with 2nd-order quasi-degenerate perturbation theory (VQDPT2), based on PES and DMS derived at the level of B3LYP-D3/cc-pVDZ. In the Scheme B (Figures 3C,D), we used a more accurate basis sets to calculate PES and DMS, B3LYP-D3/cc-pVTZ, although limited to only 8 selected coordinates: those presented in Figure 3E plus mode 70 (6–12C HOOP) and mode 136 (N-H stretch). In addition, anharmonic frequencies and intensities were obtained by VSCF with vibrational configuration interaction (VCI), more accurate but computationally more expensive than VSCF + VQDPT2.

Although the main purpose of these two anharmonic calculations was to assist us in the assignment of experimental bands to overtone and combination transitions, we first compared their performance in reproducing assignments of bands to fundamental vibrational transitions of all-*trans* PSB retinal in BR. To improve the comparison between experimental and calculated spectra, we first took into consideration that the experimental data for the BR state comes from a K-BR difference spectrum. Consequently, bands unchanged between the BR and K states are not experimentally observed, even if present in the BR state. One example are vibrations localized in the *β*-ionone ring of the retinal. For that reason, we also reconstructed anharmonic IR spectra selecting only retinal vibrational modes that we experimentally know to change between the BR and K states (Figures 3A–D, red vertical lines and blue traces).

Scheme A predicts vibrational modes 86 (10–11C), 89 (14–15C) and 93 (12–13C/14–15C) at 1,211, 1,232 cm^−1^ and at 1,269 cm^−1^, respectively (Figure 3A). The agreement with experimental bands from 10-11C, 14–15C, and 12–13C/14–15C stretches, at 1,169, 1,202 and 1,255 cm^−1^, is good, with an error of +14–30 cm^−1^ (Figure 3A). In addition, the relative areas of these bands (1/2.0/1.2) are well reproduced in the calculation (1/2.3/1.7), as collected in Table 1. The main failure of this calculation is that it assigns a tiny relative intensity to mode 92 (Figure 3A), around 10 times smaller than experimentally observed for the 8-9C stretch (see Table 1). Furthermore, it predicts the frequency of this mode to lay between those of modes 89 and 86, instead of laying between those of modes 93 and 89, as expected from the experimental data. We should note that the calculated fundamental transitions at 1,225 and 1,232 cm^−1^ are not between isolated 92 and 89 harmonic modes but between mixed contributions of both modes (see Supplementary Table S2). Regarding modes 72 and 82, their fundamental is predicted at 1,002 and at 1,040 cm^−1^, respectively (Figure 3A), very close to the experimental values for the H15C = NH HOOP (1,008 cm^−1^) and the 19C/20C methyl rock (1,050 cm^−1^). In summary, calculation of Scheme A reproduced well bands from fundamental vibrations of all-*trans* PSB retinal in the BR state, except for the 8–9C stretching (see Figure 3A and Table 1).

The commented limitation of Scheme A led us to Scheme B, more accurate but with a reduced dimensionality to compensate for the additional computational effort. The agreement between the experimental frequencies of retinal C-C stretches and those calculated for modes 86 (10–11C), 89 (14–15C) and 93 (12–13C/14–15C) was good, even though the error made (+42–50 cm^−1^) was clearly larger than for Scheme A (compare Figure 3A and Figure 3C). The calculated relative intensity of these modes (1/0.76/0.86), although reasonable, was also worse than for Scheme A (Table 1). Regarding modes 72 and 82, this calculation provided frequencies at 1,034 cm^−1^ and at 1,108 cm^−1^ (Figure 3C), off by +26 cm^−1^ and +58 cm^−1^. However, regarding mode 92 (8–9C), problematic in Scheme A, its frequency was correctly predicted to lay between modes 93 and 89, with an absolute error of +47 cm^−1^, consistent with the ∼+42–50 cm^−1^ error made for the rest of C-C modes (Figure 3C). The result indicates that the smaller basis set used in Scheme A, cc-pVDZ, is insufficient and that the larger basis set used in Scheme B, cc-pVTZ, is needed to reproduce the C-C stretching modes of π-conjugated systems. In spite this improvement, the calculated relative intensity of mode 92 was 3.5 times higher than expected from the experimental data (Figure 3C and Table 1).

Overall, both anharmonic calculations successfully reproduced frequencies and intensities of experimental bands from fundamental transitions of all-*trans* PSB retinal in the BR state. Among them, the performance of Scheme A was somehow better, but it clearly failed for mode 92 (8–9C). Scheme B, with the use of a larger basis sets, provided far more reasonable parameters for the C-C stretching vibrational modes, although with the caveat that all frequencies were systematically overestimated by ∼40–50 cm^−1^ due to the reduction of dimensionality. This indicates that for overtone and combination retinal C-C transitions, including those from mode 92, the predictions from Scheme B are likely to be more reliable than for Scheme A, although with their frequencies overestimated by ∼80–100 cm^−1^.

### Assignment of Overtones and Combinations of PSB Retinal C-C Stretches

In the 2,550–2,325 cm^−1^ region of the K-BR spectrum (Figure 2, red trace), we detect four negative bands (2,336, 2,402, 2,452 and 2,504 cm^−1^) and two positive bands (2,385 and 2,365 cm^−1^). In addition, two additional negative bands are resolved at 2,427 and 2,416 cm^−1^ after band-narrowing (Figure 2, pale red trace). A tiny negative band is also observed at around 2,478 cm^−1^. We have tentatively assigned all these bands to overtones of C-C stretches, except for bands at 2,478 cm^−1^, 2,452 cm^−1^ and 2,416 cm^−1^, assigned to combinations of C-C stretches (see Figure 2 and Table 2).

To validate and to complement these assignments, or correct them if needed, we performed two anharmonic calculations, introduced in the previous section. Figures 3B,D compares experimental and calculated spectra for all-*trans* PSB retinal in the 2,650–2,200 cm^−1^ region, containing overtone and combination modes. As explained in detail in the previous section, our anharmonic calculations overestimated fundamental νC-C transitions by ∼15–30 cm^−1^ (Scheme A) or by ∼40–50 cm^−1^ (Scheme B) and, thus, frequencies of overtones and combination bands are expected to be overestimated by 30–60 cm^−1^ (Scheme A) or by 80–100 cm^−1^ (Scheme B).

From the seven experimental negative bands, four of them are well reproduced in the calculation from Scheme A (Figure 3B and Table 2). Their frequencies are 31–60 cm^−1^ upshifted in the calculation, as expected. The experimental band at 2,336 cm^−1^, predicted at 2,395 cm^−1^, originates mostly from the overtone of mode 86 (10–11C). The experimental band at 2,402 cm^−1^, predicted at 2,462 cm^−1^, can be assigned mostly to the combination of mode 92 (8–9C) and mode 89 (14–15C), overlapping with the overtone of mode 89 (14–15C), the latter less intense. We attribute the experimental band at 2,504 cm^−1^, predicted at 2,535 cm^−1^, to the overtone of mode 93 (12–13C/14–15C). Finally, the experimental band at 2,452 cm^−1^, predicted at 2,498 cm^−1^, can be assigned mostly to a combination of mode 93 (12–13C/14–15C) and mode 89 (14–15C). See Supplementary Table S2 for a more detailed assignment of these transitions to mixed overtones/combinations of harmonic modes.

The calculation from Scheme A also displayed some apparent inconsistences with the experimental data. It predicts a rather intense transition at 2,430 cm^−1^, mostly originating from the combination of mode 89 (14–15C) and mode 86 (10–11C) (Figure 3B). However, the corresponding experimental band, expected at ∼2,390–2,370 cm^−1^, is not observed. While this could be due to an error in the calculation, we cannot completely discard that this band is present but cancelled by the more intense positive bands at 2,385 and 2,365 cm^−1^ from 13-*cis* PSB retinal in the K state (Figure 3B). A more serious issue comes by the failure of Scheme A in reproducing three experimental negative bands. One band at 2,427 cm^−1^, tentatively assigned to the overtone of the fundamental at 1,216 cm^−1^ (8–9C). The other two bands at 2,478 cm^−1^ and at 2,416 cm^−1^ (Figure 2, red spectrum), are tentatively assigned to the combination of the fundamental at 1,216 cm^−1^ (8–9C) with either the fundamental at 1,255 cm^−1^ (12–13C/14–15C) or with the fundamental at 1,201 cm^−1^ (14–15C), respectively (see Table 2). Actually, the calculation from Scheme A predicted a negligible intensity for the overtone of mode 92 (8–9C), as well as for the combination of mode 92 with mode 89 (14–15C) and for the combination of mode 92 with mode 93 (12–13C/14–15C) (Figure 3B and Table 2). It also wrongly predicted the frequency of the overtone of mode 92 at 2,450 cm^−1^, between the overtones of modes 89 and 86 (see Figure 3B). In summary, the calculation from Scheme A had problems reproducing the overtone of mode 92, as well as several of its combinations with other modes.

Scheme B reproduced all seven experimental negative bands in the 2,550–2,325 cm^−1^ region (Figure 3D). The predicted bands appear +86–106 cm^−1^ upshifted in the calculation (Figure 3D), fully consistent with the upshift of fundamental vibrations by 42–51 cm^−1^ (Figure 3C) In agreement with Scheme A, the experimental bands at 2,335, 2,402 and 2,593 cm^−1^ can be assigned to the overtones of mode 86 (10–11C), mode 89 (14–15C), and mode 93 (12–13C/14–15C), respectively (Table 2), and the experimental band at 2,452 cm^−1^ to the combination of mode 93 (12–13C/14–15C) and mode 89 (14–15C). Regarding bands not explained by the previous calculation, the weak experimental band ∼2,427 cm^−1^, predicted here at 2,529 cm^−1^ can be assigned to the overtone of mode 92 (8–9C). The nearby experimental band at 2,416 cm^−1^, with a predicted frequency at 2,519 cm^−1^ (not marked in Figure 3D by lack of space but included in Figure 2 and Table 2), can be explained by the combination of mode 92 (8–9C) and mode 89 (14–15C). Finally, the tinny experimental band at 2,478 cm^−1^, is consistent with the combination of mode 93 and mode 92, with a predicted frequency at 2,567 cm^−1^ (Table 2).

Scheme B predicts two intense vibrational transitions with no apparent experimental counterpart bands. One is, like in Scheme A, the combination of mode 89 and mode 86, predicted at 2,466 cm^−1^ (Figure 3D and Table 2). Given the systematic error in this calculation, the corresponding experimental negative band would be expected at ∼2,366–2,380 cm^−1^. The other transition is the combination of mode 92 and mode 89, at 2,478 cm^−1^ (Figure 3D and Table 2), with its experimental negative counterpart band expected at ∼2,378–2,392 cm^−1^. It is possible that these two combination bands are present but not observed in the experimental K-BR spectrum because they haven an intensity lower than calculated, being fully cancelled by overlapping positive bands from the K intermediate. Transitions involving mode 92 are likely to be weaker than calculated considering how Scheme B overestimated the relative intensity of its fundamental.

In summary, our anharmonic calculations support that four of the seven negative experimental bands between 2,550 and 2,325 cm^−1^ are overtone vibrations of C-C stretches from all-*trans* PSB retinal (Table 2). Both calculations agree that the band at 2,335 cm^−1^ corresponds to the overtone of the fundamental at 1,169 cm^−1^ (10–11C); that the band at 2,402 cm^−1^ corresponds to the overtone of the fundamental at 1,202 cm^−1^ (14–15C); and that the band at 2,504 cm^−1^ corresponds to the overtone of the fundamental at 1,255 cm^−1^ (12–13C/14–15C). The calculation from Scheme B indicates that the band at 2,427 cm^−1^ corresponds to the overtone of the fundamental at 1,217 cm^−1^ (8–9C). As for combination bands, the negative band at 2,453 cm^−1^ can be assigned to the combination of the fundamentals at 1,255 cm^−1^ (12–13C/14–15C) and at 1,202 cm^−1^ (14–15C), accordingly to both calculations. The band at 2,416 cm^−1^ corresponds to the combination of the fundamentals at 1,216 cm^−1^ (8–9C) and 1,201 cm^−1^ (14–15C), and the band at 2,478 cm^−1^ to the combination of the fundamentals at 1,255 cm^−1^ and at 1,216 cm^−1^, accordingly to the calculation from Scheme B. These assignments are collected in Table 2.

Regarding potential overtones bands from C-C stretches of 13-*cis* PSB retinal, we observe a positive band at ∼2,385 cm^−1^ and at ∼2,365 cm^−1^ in the K-BR spectrum (Figure 2, red trace). These bands are akin to the positive band at ∼1,194 cm^−1^ and the shoulder at ∼1,182 cm^−1^, but at roughly twice their wavenumber. Therefore, we assign them to overtones from 14-15C and 10–11C stretches of 13-*cis* PSB retinal (Figure 2, blue).

## Anharmonic and Coupling Mechanical Constants for PSB Retinal C-C Stretches

**FIGURE 4:**
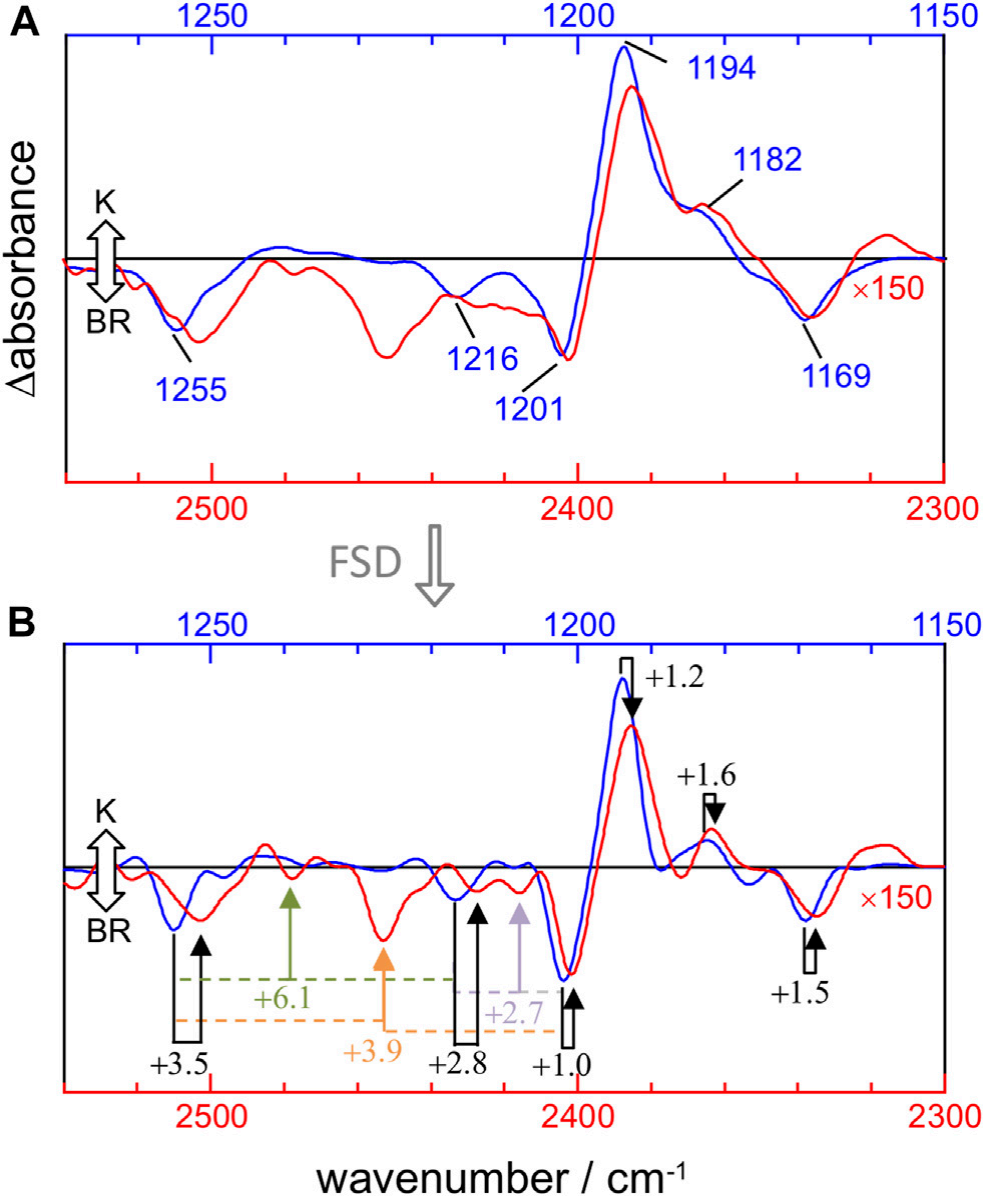
Comparison between νC-C fundamentals (blue traces) and νC-C overtones (red traces) in the K-BR spectrum **(A)** Experimental K-BR difference FT-IR spectrum in the 1,275–1,150 cm^−1^ (blue) and 2,550–2,300 cm^−1^ (red) region. Wavenumbers of bands from fundamental transitions are indicated (see Table 1 for their assignments). **(B)** Band-narrowed K-BR spectrum using a narrowing factor of 1.6 and a Lorentzian width of 7 cm^−1^ for the 1,275–1,150 cm^−1^ region and of 14 cm^−1^ for the 2,550–2,300 cm^−1^ region. Overtone bands are labelled with their estimated mechanical anharmonic constant (black) and combination bands with their mechanical coupling constant (color) constants (see Table 2 for their assignments). Note that in both **(A)** and **(B)** the overtone region is scaled by 150.

The mechanical anharmonic constant for a vibrational mode can be experimentally determined as the fundamental minus half the first overtone frequency. On the other hand, the mechanical coupling constant between two vibrational modes is given by the sum of their fundamentals minus their combination frequency. To visualize these constants, we overlayed the 1,270–1,150 cm^−1^ and 2,540–2,300 cm^−1^ regions of the K-BR difference spectrum, before (Figure 4A) and after (Figure 4B) band-narrowing. To quantify more accurately the anharmonic constants, we determined the frequency of fundamental, combination and overtone bands from band-narrowed K-BR spectra (shown in Figure 4B and collected in Table 2 for the negative bands).

For the 14–15C and 10–11C vibrations of all-*trans* PSB retinal, with fundamentals at ∼1,201 and ∼1,169 cm^−1^ (Figure 4A), respectively, the mechanical anharmonic constants are +1.0 cm^−1^ and +1.5 cm^−1^ (Figure 4B). For 13-*cis* PSB retinal in the K intermediate the coupled 14–15C and 10–11C vibrations, with fundamentals at 1,194 and 1,182 cm^−1^, display mechanical anharmonic constants of 1.2 cm^−1^ and 1.6 cm^−1^, respectively (Figure 4A, right). Thus, the mechanical anharmonic constant for the 14–15C and 10–11C stretches are quite low, regardless of the isomerization state of the PSB retinal: 1.0–1.5 cm^−1^ in the BR state (all-*trans*), and 1.2–1.6 cm^−1^ in K intermediate (13-*cis*).

Returning to all-*trans* PSB retinal, the vibrational mode containing both 12–13C and 14–15C contributions, with the fundamental at ∼1,255 cm^−1^ (Figure 4A), displays a mechanical anharmonic constant of +3.5 cm^−1^ (Figure 4B). For the C8-C9 stretching, contributing to the fundamental at 1,216 cm^−1^, its mechanical anharmonic constant is +2.8 cm^−1^ (Figure 4B). Regarding mechanical coupling constants, the one between the fundamental at 1,201 cm^−1^ (14–15C) and at 1,255 cm^−1^ (12–13C/14–15C) is of +3.9 cm^−1^ (Figure 4B); between the fundamental at 1,201 cm^−1^ (14–15C) and at 1,216 cm^−1^ (8–9C) of +2.7 cm^−1^; and between the fundamental at 1,255 cm^−1^ (12–13C/14–15C) and at 1,216 cm^−1^ (8–9C) of +6.1 cm^−1^.

The mechanical anharmonic and coupling constants for all-*trans* PSB retinal determined from Scheme A, estimated using the same approach applied to the experimental data, are in reasonable agreement with those determined experimentally (Table 2). In particularly, the anharmonic constant for the of mode 86 (10–11C) was calculated to be +0.9 cm^−1^, comparing well with the experimental value + 1.5 cm^−1^. For other transitions the error was somehow larger: for mode 89 (14–15C) and for mode 93 (12–13C/14–15C) the mechanical anharmonic constant was calculated to be +3.9 cm^−1^ and +1.1 cm^−1^, but experimentally determined to be of +1.0 cm^−1^ and +3.5 cm^−1^, respectively (Table 2). The coupling constant for the combination of modes 89 (14–15C) and 93 (12–13C/14–15C) was calculated to be +2.1 cm^−1^, not far from its experimental value of +3.9 cm^−1^ (Table 2).

Regarding mechanical anharmonic constants for Scheme B, they were very small, as the experimental ones, but negative instead of positive. They ranged from –1.8 (mode 89) to –0.1 cm^−1^ (mode 86), as collected in Table 2. The mechanical coupling constants were, likewise, predicted to be small and negative, ranging from –4.4 to –3.1 cm^−1^ (Table 2).

### Width and Relative Intensity of Bands From Overtone Transitions of C-C Stretches

Figure 4A compares the fundamental and overtone/combination region of C-C stretches of the retinal. These two regions were scaled to make the band at 1,169 cm^−1^ (10–11C) and its overtone at 2,336 cm^−1^ to be of roughly the same intensity. Thus, it can be visually appreciated that the overtones of retinal C-C stretches are roughly 150 times weaker in intensity than their fundamentals. More relevant are area ratios between fundamental and overtones, directly comparable with the intensities in the calculations. These ratios are 60, 110, 80, 85, 70, and 50 for the fundamental vibrations at 1,255 (–), 1,216 (–), 1,201 (–), 1,194 (+), 1,182 (+) and 1,169 (–) cm^−1^, respectively (Table 2). In contrast, overtones were calculated to be between 250 and 1800 weaker than their corresponding fundamental transitions in Scheme A, and between 240 and 1700 in Scheme B (Table 2). Thus, C-C overtones are ∼4–20 more intense in the experiments than in our anharmonic calculations, a notable difference that calls for an explanation. To clarify this point, we have analyzed the magnitude of the first and second-order derivatives of the dipole moment with respect to C-C stretching modes, as detailed in the Supplementary Information (see Supplementary Data section). Briefly, to reproduce the experimental relative area between fundamental and overtone bands, the ratio between the magnitude of the second derivative and the first derivative of the dipole moment should be between 2 and 6 times larger than in our calculations, i.e., DFT calculations at the level of B3LYP-D3/cc-pVTZ underestimated the electrical anharmonicities by a factor of 2–6.

Figure 4A also informs us that overtone bands from C-C vibrations of the PSB retinal are roughly twice broader than fundamentals bands, except for the overtone of the 1,255 cm^−1^ fundamental, which appears 3–4 times broader. Thus, although the separation of bands is twice larger for overtones than for fundamental vibrations, this is of no help to improve their band resolution, particularly when considering that overtones bands co-exist in this region with combination bands.

### Combination Bands of Retinal C-C Stretches With CH_3_ Rocks and HOOPs

The negative band at 1,008 cm^−1^ corresponds to the symmetric in-plane rocking combination of 19C and 20C methyl groups from the BR state (Smith et al., 1987; Maeda et al., 1995), akin to mode 72 in our calculations (Table 1). Scheme A predicts the combination of mode 72 with the three more intense C-C modes of all-*trans* PSB retinal (modes 93, 89 and 86), giving rise to combination bands at 2,270, 2,235, and 2,202 cm^−1^ (Table 2). This assignment agrees with the experimental negative bands at 2,257 and 2,212 and 2,176 cm^−1^, which can be explained by the combination of the fundamental at 1,008 cm^−1^ with fundamental transitions from C-C stretches at 1,255, 1,202 and 1,169 cm^−1^, respectively. According to the above assignment, the experimental mechanical coupling constants between the methyl rock and these C-C stretches are +6.2, –1.8 and +1.4 cm^−1^, respectively. The prediction from Scheme B is similar, except for the fact that mode 72 is predicted to couple with mode 92 instead of with mode 89. Accepting this last assignment, the experimental band at 2,212 cm^−1^ would result from the combination of the fundamentals at 1,008 cm^−1^ and at 1,216 cm^−1^, with a mechanical coupling constant of +13 cm^−1^. Between these two possibilities, we think that in this case it is more reasonable to assignment the band at 2,212 cm^−1^ to the combination of the fundamentals at 1,008 cm^−1^ and 1,201 cm^−1^ (modes 72 and 89), as suggested by Scheme A.

Positive bands at 2,165, 2,149 and 2,135 cm^−1^ fit well with the combination of the 14–15C + 10–11C stretch at 1,194 cm^−1^ with in-phase HOOP bands at 974, 956 and 941 cm^−1^ (Figure 2). In spite the high relative intensity of these combination bands, the derived mechanical coupling constants are very small, between –0.2 and +1.0 cm^−1^.

### Unassigned Bands Between 2,100 and 1,850 cm^−1^

A broad negative and a positive band are resolved at around 2,070 (–) cm^−1^ and around at 2,000 (+) cm^−1^, respectively, with a fine structure revealed after band-narrowing by FSD (Figure 2, bottom). Given their frequency, they might arise from combination bands between out-of-phase HOOP bands, located between 850 and 750 cm^−1^ (Smith et al., 1987), and C-C stretches. Relatively narrow alternating positive and negative bands are observed between 1,950 and 1,850 cm^−1^. Although from their frequency they could be overtones of in-phase HOOP bands, they do not display any clear resemblance to HOOPs bands from fundamental transitions. Alternatively, these bands could also originate from the coupling of C-C stretching vibrations with some fundamental vibrations at around 700 cm^−1^. Experiments with isotope labelled retinal will be needed for the assignment of bands in this spectral region to specific overtones/combination vibrational transitions.

## General Discussion

Bands in the mid-IR region of proteins are, by default, often assumed to corresponds to fundamental transitions from molecular vibrations. While this assumption is generally correct, in this work we have shown that the light-induced FT-IR difference spectrum of bacteriorhodopsin contains bands in the mid-IR region coming not from fundamental but from overtone and combination transitions. In particular, we have shown that bands between 2,540 and 2,300 cm^−1^ originate from overtone and combination bands from retinal C-C vibrations, and bands between 2,300 and 2,100 cm^−1^ result from transitions involving the combination of retinal C-C vibrations with either methyl rocks or with hydrogen-out-of-plane vibrations.

Another notable observation of the present work is the fact that retinal C-C stretching modes are, apparently, close to be mechanically harmonic, with mechanical anharmonic constants for the 14–15C and 10–11C stretches between 1.0 and 1.6 cm^−1^ for both all-*trans* PSB and 13-*cis* PSB. Despite these small values, the overtones from 14-15C and 10–11C stretches display areas only ∼50–85 smaller than their corresponding fundamentals. To have a reference, the anharmonic constant for CO bound to myoglobin is 12–14 cm^−1^, almost 10 times larger than for the retinal 14–15C and 10–11C stretches. Yet, the area of this overtone band is ∼100 smaller than for its fundamental band (Sage et al., 2011). To give an explanation of why the overtone bands from the retinal C-C stretches are intense in spite their low mechanical anharmonicity, we have to note that the intensity of overtone bands depends both on the mechanical anharmonic constant (through the wavefunction overlap of the fundamental and the second excited vibrational state) and on the electrical anharmonicity (related to $\partial^{2}\mu /\partial Q^{2}$ , where **Q** is the mode coordinate vector and **μ** is the molecular dipole moment vector) (Sandorfy et al., 2006). Thus, it is reasonable to conclude that the rather high intensity of the overtone bands indicates that the electrical anharmonicity of the retinal C-C stretches is unusually large. We demonstrated that in our most accurate calculation (Scheme B) the electrical anharmonicity accounted for ∼60% of the overtone intensity (see the Supplementary Data and Supplementary Table S1 for details). Because our calculations reproduced mechanical anharmonicities but notably underestimated the experimental intensities of the overtone bands, it is likely that this percentage is even larger in the experimental data.

We wonder how similar are mechanical anharmonic constants and fundamental/overtone area ratios for the C-C stretches from the PSB retinal in BR when compared to C-C stretches from other molecules. This question is difficult to answer though. The reason is that C-C stretches have a very low transition dipole moment and, thus, very weak intensities for fundamental (and overtone) transitions. Indeed, we have not been able to find examples in the literature for experimental mechanical anharmonic constants of C-C stretches or fundamental/overtone area ratios. The case of PSB retinal is peculiar. Because of the π-conjugated system, when the SB is protonated the positive charge is delocalized along the polyene chain, although with a gradient: the positive charge is highest at the N atom, decreasing in the direction to the ring (Babitzki et al., 2009). As a result, the C-C (and C=C) bonds are polarized and their stretching vibrations is associated with a relatively large change in the dipole moment, giving IR intensity to fundamental retinal C-C transitions (Babitzki et al., 2009). In agreement, the experimental IR intensity of the C-C stretching vibrations of the PSB retinal drops dramatically when deprotonated, as in the M intermediate of BR (Maeda, 1995).

We believe that the charge delocalization in the PSB retinal explains not only the large transition dipole moment of the C-C stretching vibrations (i.e., the unusually high IR intensity for the fundamental transitions), but accounts as well the high electrical anharmonicity of the C-C stretches (i.e., the unusually high overtone IR intensity). If this interpretation is correct, overtone and combination bands from C-C stretches will be sensitive not only to the retinal conformation but also to the charge distribution of the PSB retinal (Babitzki et al., 2009) and, thus, to the environment and nature of the binding pocket of the PSB retinal. In this respect, it is interesting to note the similar but not identical anharmonic mechanical constants and fundamental/overtone area ratio for positive and negative bands in the K-BR FT-IR difference spectrum. This observation indicates that the mechanical and electrical anharmonicity of retinal C-C stretches might differ after retinal isomerization.

The present work also provides information for mechanical coupling constants between vibrational modes. We found that coupling constants between C-C stretches are between +2.7 and +6.1 cm^−1^. Coupling constants between C-C stretches and C-CH_3_ (methyl rocks) or C-H wags (HOOP vibrations) of the retinal are even smaller, in most cases in the order or lower than 2 cm^−1^. The observation of intense combination bands between the C-C and HOOP vibrations of the retinal in the K intermediate, in spite their coupling constant being smaller than 1 cm^−1^, indicate that both vibrations are electrically coupled. In other words, $\partial^{2}\mu /\partial Q_{i}Q_{j}\neq 0$ , meaning that oscillations along a C-C coordinate affect how the dipole moment changes along a HOOP vibration coordinate, and *vice versa*, resulting in their coupling. An interesting question is whether the electrical coupling between C-C and HOOP vibrations could allow to transfer energy from one vibrational mode to the other, and if this transfer could help or play a role in the thermal relaxation of the retinal during its photoisomerization from all-*trans* to 13-*cis*.

The in-phase C=C stretching vibration of PSB retinal in the BR, at 1,526 cm^−1^, has been shown by 2D-IR spectroscopy to have a mechanical anharmonic constant of 7 cm^−1^ (Andresen and Hamm, 2009), substantially larger than the values we determined here for the C-C stretches. Why are the C-C stretches mechanically more harmonic than the C=C stretches? One difference between both types of vibrations is that the modes assigned to C-C stretches also involve in-plane bending models of the hydrogen atoms attached to the polyene chain (CCH in-plane rocks), as revealed by normal mode calculations and supported by ^13^C and ^2^H labelling (Smith et al., 1987; Babitzki et al., 2009). Thus, it is possible that the relatively low mechanical anharmonicity of the C-C stretches could be, at least partially, caused by the contributions from these bending modes to the vibrational modes, which are expected to behave more harmonically than stretching vibrations. A way to test this hypothesis in the future could be experiments with perdeuterated retinal, uncoupling the C-C stretches from in-plane bending modes.

After introducing overtones and combinations C-C stretching bands from the PSB retinal in the BR and K states, further work should explore the potential utility of these bands. For instance, resolving overtone and combination bands for the rest of the intermediates in the photocycle of BR, something we are already working on, might provide information about how much sensitive overtone/combination bands are than fundamental bands to conformation and environment changes of the retinal. We should also note that the present results on overtone and combination bands have been limited to 80 K. By extending these experiments to other temperatures, we should be able to use the potentially higher sensitivity of overtones/combination bands to reveal how the conformation/environment of all-*trans* PSB retinal in the BR is affected by the temperature at which the photocycle is initiated. A final question requiring further investigation is the role of the environment provided by the retinal binding pocket of BR on the low mechanical and high electrical anharmonicity of the PSB retinal, if any. In this respect it would be desirable to resolve and characterize, as a comparison, overtone and combination bands of PSB all-*trans* retinal in solution. In the past we have studied a chemical model for PSB retinal in solution by UV-vis spectroscopy (Lórenz-Fonfría et al., 2010), but so far we have not succeeded in conducting similar studies by FT-IR spectroscopy.

## Data Availability

The datasets presented in this study can be found in online repositories. The names of the repository/repositories and accession number(s) can be found below: Mendeley Data (https://data.mendeley.com/datasets/n5t4jh5cm9/1).

## References

[B1] Adhikary R. Zimmermann J. Romesberg F. E. (2017). Transparent Window Vibrational Probes for the Characterization of Proteins with High Structural and Temporal Resolution. Chem. Rev. 117, 1927–1969. 10.1021/acs.chemrev.6b00625 28106985

[B2] Andresen E. R. Hamm P. (2009). Site-Specific Difference 2D-IR Spectroscopy of Bacteriorhodopsin. J. Phys. Chem. B 113, 6520–6527. 10.1021/jp810397u 19358550

[B3] Babitzki G. Mathias G. Tavan P. (2009). The Infrared Spectra of the Retinal Chromophore in Bacteriorhodopsin Calculated by a DFT/MM Approach. J. Phys. Chem. B 113, 10496–10508. 10.1021/jp902432e 19580300

[B4] Bagley K. Dollinger G. Eisenstein L. Singh A. K. Zimányi L. (1982). Fourier Transform Infrared Difference Spectroscopy of Bacteriorhodopsin and its Photoproducts. Proc. Natl. Acad. Sci. 79, 4972–4976. 10.1073/pnas.79.16.4972 6956906 PMC346807

[B5] Balashov S. P. Ebrey T. G. (2007). Trapping and Spectroscopic Identification of the Photointermediates of Bacteriorhodopsin at Low Temperatures. Photochem. Photobiol. 73, 453–462. 10.1562/0031-8655(2001)0730453TASIOT2.0.CO2 11367564

[B6] Barth A. Zscherp C. (2002). What Vibrations Tell about Proteins. Quart. Rev. Biophys. 35, 369–430. 10.1017/S0033583502003815 12621861

[B7] Becke A. D. (1993). Density‐functional Thermochemistry. III. The Role of Exact Exchange. J. Chem. Phys. 98, 5648–5652. 10.1063/1.464913

[B8] Best R. B. Zhu X. Shim J. Lopes P. E. M. Mittal J. Feig M. (2012). Optimization of the Additive CHARMM All-Atom Protein Force Field Targeting Improved Sampling of the Backbone ϕ, ψ and Side-Chain χ_1_ and χ_2_ Dihedral Angles. J. Chem. Theor. Comput. 8, 3257–3273. 10.1021/CT300400X PMC3549273 23341755

[B9] Braiman M. S. Mogi T. Marti T. Stern L. J. Khorana H. G. Rothschild K. J. (1988). Vibrational Spectroscopy of Bacteriorhodopsin Mutants: Light-Driven Proton Transport Involves Protonation Changes of Aspartic Acid Residues 85, 96, and 212. Biochemistry 27, 8516–8520. 10.1021/bi00423a002 2851326

[B10] Brooks B. R. Brooks C. L. Mackerell A. D. Nilsson L. Petrella R. J. Roux B. (2009). CHARMM: The Biomolecular Simulation Program. J. Comput. Chem. 30, 1545–1614. 10.1002/jcc.21287 19444816 PMC2810661

[B11] Domratcheva T. Hartmann E. Schlichting I. Kottke T. (2016). Evidence for Tautomerisation of Glutamine in BLUF Blue Light Receptors by Vibrational Spectroscopy and Computational Chemistry. Sci. Rep. 6, 22669. 10.1038/srep22669 26947391 PMC4780082

[B12] Dunning T. H. (1989). Gaussian Basis Sets for Use in Correlated Molecular Calculations. I. The Atoms boron through Neon and Hydrogen. J. Chem. Phys. 90, 1007–1023. 10.1063/1.456153

[B13] Earnest T. N. Roepe P. Braiman M. S. Gillespie J. Rothschild K. J. (1986). Orientation of the Bacteriorhodopsin Chromophore Probed by Polarized Fourier Transform Infrared Difference Spectroscopy. Biochemistry 25, 7793–7798. 10.1021/bi00372a002 3801443

[B14] Ernst O. P. Lodowski D. T. Elstner M. Hegemann P. Brown L. S. Kandori H. (2014). Microbial and Animal Rhodopsins: Structures, Functions, and Molecular Mechanisms. Chem. Rev. 114, 126–163. 10.1021/cr4003769 24364740 PMC3979449

[B15] Fahmy K. Siebert F. Tavan P. (1991). Structural Investigation of Bacteriorhodopsin and Some of its Photoproducts by Polarized Fourier Transform Infrared Spectroscopic Methods-Difference Spectroscopy and Photoselection. Biophys. J. 60, 989–1001. 10.1016/S0006-3495(91)82136-1 19431812 PMC1260156

[B16] Fischer W. B. Sonar S. Marti T. Khorana H. G. Rothschild K. J. (1994). Detection of a Water Molecule in the Active-Site of Bacteriorhodopsin: Hydrogen Bonding Changes during the Primary Photoreaction. Biochemistry 33, 12757–12762. 10.1021/bi00209a005 7947680

[B17] Frisch M. J. Trucks G. W. Schlegel H. B. Scuseria G. E. Robb M. A. Cheeseman J. R. (2016). Gaussian 16, Revision C.01. Wallingford, CT: Gaussian Inc.

[B18] Gerwert K. Siebert F. (1986). Evidence for Light-Induced 13-*cis* , 14-s-*cis* Isomerization in Bacteriorhodopsin Obtained by FTIR Difference Spectroscopy Using Isotopically Labelled Retinals. EMBO J. 5, 805–811. 10.1002/j.1460-2075.1986.tb04285.x 16453681 PMC1166862

[B19] Ghosh A. Ostrander J. S. Zanni M. T. (2017). Watching Proteins Wiggle: Mapping Structures with Two-Dimensional Infrared Spectroscopy. Chem. Rev. 117, 10726–10759. 10.1021/acs.chemrev.6b00582 28060489 PMC5500453

[B20] Grimme S. Antony J. Ehrlich S. Krieg H. (2010). A Consistent and Accurate ab initio Parametrization of Density Functional Dispersion Correction (DFT-D) for the 94 Elements H-Pu. J. Chem. Phys. 132, 154104. 10.1063/1.3382344 20423165

[B21] Groh W. (1988). Overtone Absorption in Macromolecules for Polymer Optical Fibers. Makromol. Chem. 189, 2861–2874. 10.1002/MACP.1988.021891213

[B22] Hayashi S. Tajkhorshid E. Kandori H. Schulten K. (2004). Role of Hydrogen-Bond Network in Energy Storage of Bacteriorhodopsin's Light-Driven Proton Pump Revealed by Ab Initio Normal-Mode Analysis. J. Am. Chem. Soc. 126, 10516–10517. 10.1021/ja047506s 15327290

[B23] Ito S. Kandori H. Lorenz-Fonfria V. A. (2018). Potential Second-Harmonic Ghost Bands in Fourier Transform Infrared (FT-IR) Difference Spectroscopy of Proteins. Appl. Spectrosc. 72, 956–963. 10.1177/0003702818757521 29350538

[B24] Jorgensen W. L. Chandrasekhar J. Madura J. D. Impey R. W. Klein M. L. (1983). Comparison of Simple Potential Functions for Simulating Liquid Water. J. Chem. Phys. 79, 926–935. 10.1063/1.445869

[B25] Jung J. Mori T. Kobayashi C. Matsunaga Y. Yoda T. Feig M. (2015). GENESIS: a Hybrid-Parallel and Multi-Scale Molecular Dynamics Simulator with Enhanced Sampling Algorithms for Biomolecular and Cellular Simulations. WIREs Comput. Mol. Sci. 5, 310–323. 10.1002/WCMS.1220 PMC4696414 26753008

[B26] Kandori H. Kinoshita N. Shichida Y. Maeda A. (1998). Protein Structural Changes in Bacteriorhodopsin upon Photoisomerization as Revealed by Polarized FTIR Spectroscopy. J. Phys. Chem. B 102, 7899–7905. 10.1021/jp981949z

[B27] Kandori H. (2020). Structure/Function Study of Photoreceptive Proteins by FTIR Spectroscopy. Bull. Chem. Soc. Jpn. 93, 904–926. 10.1246/bcsj.20200109

[B28] Kauppinen J. K. Moffatt D. J. Mantsch H. H. Cameron D. G. Spectroscopy R. (1981). Fourier Self-Deconvolution: A Method for Resolving Intrinsically Overlapped Bands. Appl. Spectrosc. 35, 271–276. 10.1366/0003702814732634

[B29] Kobayashi C. Jung J. Matsunaga Y. Mori T. Ando T. Tamura K. (2017). GENESIS 1.1: A Hybrid-Parallel Molecular Dynamics Simulator with Enhanced Sampling Algorithms on Multiple Computational Platforms. J. Comput. Chem. 38, 2193–2206. 10.1002/JCC.24874 28718930

[B30] Kötting C. Gerwert K. (2013). Monitoring Protein-Ligand Interactions by Time-Resolved FTIR Difference Spectroscopy. Methods Mol. Biol. 1008, 299–323. 10.1007/978-1-62703-398-5_11 23729257

[B31] Kottke T. Lórenz-Fonfría V. A. Heberle J. (2017). The Grateful Infrared: Sequential Protein Structural Changes Resolved by Infrared Difference Spectroscopy. J. Phys. Chem. B 121, 335–350. 10.1021/acs.jpcb.6b09222 28100053

[B32] Koziol K. L. Johnson P. J. Stucki-Buchli B. Waldauer S. A. Hamm P. (2015). Fast Infrared Spectroscopy of Protein Dynamics: Advancing Sensitivity and Selectivity. Curr. Opin. Struct. Biol. 34, 1–6. 10.1016/J.SBI.2015.03.012 25900180

[B33] Lee C. Yang W. Parr R. G. (1988). Development of the Colle-Salvetti Correlation-Energy Formula into a Functional of the Electron Density. Phys. Rev. B 37, 785–789. 10.1103/PhysRevB.37.785 9944570

[B34] Lórenz-Fonfría V. A. Furutani Y. Ota T. Ido K. Kandori H. (2010). Protein Fluctuations as the Possible Origin of the thermal Activation of Rod Photoreceptors in the Dark. J. Am. Chem. Soc. 132, 5693–5703. 10.1021/ja907756e 20356096

[B35] Lorenz-Fonfria V. A. (2020). Infrared Difference Spectroscopy of Proteins: From Bands to Bonds. Chem. Rev. 120, 3466–3576. 10.1021/acs.chemrev.9b00449 32202114

[B36] Luecke H. Schobert B. Richter H.-T. Cartailler J.-P. Lanyi J. K. (1999). Structure of Bacteriorhodopsin at 1.55 Å Resolution. J. Mol. Biol. 291, 899–911. 10.1006/jmbi.1999.3027 10452895

[B37] Maeda A. (1995). Application of FTIR Spectroscopy to the Structural Study on the Function of Bacteriorhodopsin. Isr. J. Chem. 35, 387–400. 10.1002/ijch.199500038

[B38] Maeda A. Sasaki J. Pfefferlé J.-M. Shichida Y. Yoshizawa T. (1991). Fourier Transform Infrared Spectral Studies on the Schiff Base Mode of All-Trans Bacteriorhodopsin and its Photointermediates, K and L. Photochem. Photobiol. 54, 911–921. 10.1111/j.1751-1097.1991.tb02111.x

[B39] Maeda A. Yamazaki Y. Sasaki J. Hatanaka M. Kandori H. Needleman R. (1995). Interaction of Tryptophan-182 with the Retinal 9-methyl Group in the L Intermediate of Bacteriorhodopsin. Biochemistry 34, 577–582. 10.1021/bi00002a024 7819252

[B40] Nakamura S. Noguchi T. (2017). Infrared Determination of the Protonation State of a Key Histidine Residue in the Photosynthetic Water Oxidizing Center. J. Am. Chem. Soc. 139, 9364–9375. 10.1021/jacs.7b04924 28635275

[B41] Nakamura S. Noguchi T. (2016). Quantum Mechanics/molecular Mechanics Simulation of the Ligand Vibrations of the Water-Oxidizing Mn_4_CaO_5_ Cluster in Photosystem II. Proc. Natl. Acad. Sci. USA 113, 12727–12732. 10.1073/pnas.1607897113 27729534 PMC5111704

[B42] Neutze R. Pebay-Peyroula E. Edman K. Royant A. Navarro J. Landau E. M. (2002). Bacteriorhodopsin: a High-Resolution Structural View of Vectorial Proton Transport. Biochim. Biophys. Acta Biomembr. 1565, 144–167. 10.1016/S0005-2736(02)00566-7 12409192

[B43] Oesterhelt D. Stoeckenius W. (1974). Isolation of the Cell Membrane of Halobacterium Halobium and its Fractionation into Red and Purple Membrane. Methods Enzymol. 31, 667–678. 10.1016/0076-6879(74)31072-5 4418026

[B44] Olsson M. H. M. Søndergaard C. R. Rostkowski M. Jensen J. H. (2011). PROPKA3: Consistent Treatment of Internal and Surface Residues in Empirical pKa Predictions. J. Chem. Theor. Comput. 7, 525–537. 10.1021/ct100578z 26596171

[B45] Panek P. T. Jacob C. R. (2016). Anharmonic Theoretical Vibrational Spectroscopy of Polypeptides. J. Phys. Chem. Lett. 7, 3084–3090. 10.1021/acs.jpclett.6b01451 27472016

[B46] Peuker S. Andersson H. Gustavsson E. Maiti K. S. Kania R. Karim A. (2016). Efficient Isotope Editing of Proteins for Site-Directed Vibrational Spectroscopy. J. Am. Chem. Soc. 138, 2312–2318. 10.1021/jacs.5b12680 26796542

[B47] Rothschild K. J. Marrero H. Braiman M. Mathies R. (1984). Primary Photochemistry of Bacteriorhodopsin: Comparison of Fourier Transform Infrared Difference Spectra with Resonance Raman Spectra. Photochem. Photobiol. 40, 675–679. 10.1111/j.1751-1097.1984.tb05359.x 6514815

[B48] Rothschild K. J. Marrero H. (1982). Infrared Evidence that the Schiff Base of Bacteriorhodopsin Is Protonated: bR570 and K Intermediates. Proc. Natl. Acad. Sci. 79, 4045–4049. 10.1073/pnas.79.13.4045 6955790 PMC346573

[B49] Sage J. T. Zhang Y. McGeehan J. Ravelli R. B. G. Weik M. van Thor J. J. (2011). Infrared Protein Crystallography. Biochim. Biophys. Acta Proteins Proteomics 1814, 760–777. 10.1016/J.BBAPAP.2011.02.012 21376143

[B50] Šali A. (2014). MODELLER: A Program for Protein Structure Modeling. Release 9.14. Available at: http://salilab.org/modeller/ . (Accessed December 8, 2019).

[B51] Sandorfy C. Buchet R. Lachenal G. (2006). “Principles of Molecular Vibrations for Near-Infrared Spectroscopy,” in Near-Infrared Spectroscopy in Food Science and Technology (Hoboken, NJ, USA: John Wiley & Sons), 11–46. 10.1002/9780470047705.ch2

[B52] Siebert F. Mäntele W. (1983). Investigation of the Primary Photochemistry of Bacteriorhodopsin by Low-Temperature Fourier-Transform Infrared Spectroscopy. Eur. J. Biochem. 130, 565–573. 10.1111/j.1432-1033.1983.tb07187.x 6825710

[B53] Smith S. O. Braiman M. S. Myers A. B. Pardoen J. A. Courtin J. M. L. Winkel C. (1987). Vibrational Analysis of the All-Trans-Retinal Chromophore in Light-Adapted Bacteriorhodopsin. J. Am. Chem. Soc. 109, 3108–3125. 10.1021/ja00244a038

[B54] Struve W. S. (1989). Fundamentals of Molecular Spectroscopy. New York, NY: Wiley.

[B55] Weidlich O. Siebert F. (1993). Time-Resolved Step-Scan FT-IR Investigations of the Transition from KL to L in the Bacteriorhodopsin Photocycle: Identification of Chromophore Twists by Assigning Hydrogen-Out-Of-Plane (HOOP) Bending Vibrations. Appl. Spectrosc. 47, 1394–1400. 10.1366/0003702934067351

[B56] Weidlich O. Ujj L. Jäger F. Atkinson G. H. (1997). Nanosecond Retinal Structure Changes in K-590 during the Room-Temperature Bacteriorhodopsin Photocycle: Picosecond Time-Resolved Coherent Anti-stokes Raman Spectroscopy. Biophys. J. 72, 2329–2341. 10.1016/S0006-3495(97)78877-5 9129836 PMC1184428

[B57] Welke K. Watanabe H. C. Wolter T. Gaus M. Elstner M. (2013). QM/MM Simulations of Vibrational Spectra of Bacteriorhodopsin and Channelrhodopsin-2. Phys. Chem. Chem. Phys. 15, 6651. 10.1039/c3cp44181d 23385325

[B58] Wickstrand C. Nogly P. Nango E. Iwata S. Standfuss J. Neutze R. (2019). Bacteriorhodopsin: Structural Insights Revealed Using X-Ray Lasers and Synchrotron Radiation. Annu. Rev. Biochem. 88, 59–83. 10.1146/annurev-biochem-013118-111327 30830799

[B59] Yagi K. Hirao K. Taketsugu T. Schmidt M. W. Gordon M. S. (2004). Ab Initiovibrational State Calculations with a Quartic Force Field: Applications to H_2_CO, C_2_H_4_, CH_3_OH, CH_3_CCH, and C_6_H_6_ . J. Chem. Phys. 121, 1383–1389. 10.1063/1.1764501 15260682

[B60] Yagi K. Hirata S. Hirao K. (2008). Vibrational Quasi-Degenerate Perturbation Theory: Applications to Fermi Resonance in CO_2_, H_2_CO, and C_6_H_6_ . Phys. Chem. Chem. Phys. 10, 1781–1788. 10.1039/B719093J 18350183

[B61] Yagi K. Ito S. Sugita Y. (2021). Exploring the Minimum-Energy Pathways and Free-Energy Profiles of Enzymatic Reactions with QM/MM Calculations. J. Phys. Chem. B 125, 4701–4713. 10.1021/acs.jpcb.1c01862 33914537 PMC10986901

[B62] Yagi K. Keçeli M. Hirata S. (2012). Optimized Coordinates for Anharmonic Vibrational Structure Theories. J. Chem. Phys. 137, 204118. 10.1063/1.4767776 23205992

[B63] Yagi K. Otaki H. (2014). Vibrational Quasi-Degenerate Perturbation Theory with Optimized Coordinates: Applications to Ethylene and Trans-1,3-butadiene. J. Chem. Phys. 140, 084113. 10.1063/1.4866365 24588154

[B64] Yagi K. Taketsugu T. Hirao K. Gordon M. S. (2000). Direct Vibrational Self-Consistent Field Method: Applications to H_2_O and H_2_CO. J. Chem. Phys. 113, 1005–1017. 10.1063/1.481881

[B65] Yagi K. Yamada K. Kobayashi C. Sugita Y. (2019). Anharmonic Vibrational Analysis of Biomolecules and Solvated Molecules Using Hybrid QM/MM Computations. J. Chem. Theor. Comput. 15, 1924–1938. 10.1021/acs.jctc.8b01193 PMC8864611 30730746

