# Supplementary material for: Retinal Vibrations in Bacteriorhodopsin are Mechanically Harmonic but Electrically Anharmonic: Evidence From Overtone and Combination Bands
Source: Front Mol Biosci. 2021 Dec 17;8:749261. doi: 10.3389/fmolb.2021.749261 (PMC8718751; doi:10.3389/fmolb.2021.749261)
Supplement: Supplementary file 1 [file DataSheet1.PDF]

Supplementary Material

1 Supplementary Data

1.1 Fundamental and overtone intensities

In order to clarify the contribution of mechanical and electrical anharmonicity to the IR intensity of overtone/combination transitions from the retinal in BR we used Scheme C (see Materials and Methods in the main text). Scheme C was based on Scheme B but using harmonic oscillator wavefunctions, retaining the full dipole moment surfaces. Thus, contrary to Scheme B, in Scheme C all the IR intensity from overtone and combination bands must originate from electrical anharmonicities. Comparison of the results from Scheme B and C are presented in the Supplementary Table 1. We compared the calculated intensity of fundamental, overtone and combination transitions between Scheme C and Scheme B as the I_C/I_B ratio. This ratio ranged between 0.2 and 1.2 for overtone and combination retinal C-C stretches, with an average value of 0.65. In other words, more than half of the intensity of overtone and combination bands calculated in Scheme B can be ascribed to electrical anharmonicity. One outlier is the overtone of 8C-9C (mode 92), where I_C/I_B is as small as 0.2, meaning that in our calculations most of its intensity can be ascribed to mechanical anharmonicity.

The Supplementary Fig. 6 shows the variation of the x, y and z components of dipole moment as a function of C-C stretching modes for scheme C (and scheme B). Note that the C-C bonds of retinal were oriented more or less along the y-axis in the crystal structure, 1C3W (Fig. S2), so that the y-component had the largest variation in the dipole moment. By fitting the profile of the dipole moment to a second-order polynomial, we obtained the first (a_x, a_y, a_z) and second-order (b_x, b_y, b_z) coefficients, respectively. In the mechanically harmonic approximation, the overtone intensity is proportional to the second-order coefficients as:

$$I_{ovtn} \propto \left(\frac{\hbar}{\omega}\right)^2 \left(b_x^2 + b_y^2 + b_z^2\right).$$

where $\hbar$ is the reduced Plank constant and ω is the harmonic frequency. The non-zero second-order coefficients, in the order of 10^{-5} to 10^{-6} (in atomic units) in our calculations (see Supplementary Figure 7), is responsible for the overtone intensity. Note that the intensity of the fundamental is derived in a similar way, but depends on the first order coefficients, as:

$$I_{fund} \propto \frac{\hbar}{2\omega} \left(a_x^2 + a_y^2 + a_z^2\right).$$

The ratio I_{fund}/I_{ovtn} obtained using the above equations is given by:

$$R = \frac{I_{fund}}{I_{ovtn}} = \frac{\omega}{2\hbar}\left(a_x^2 + a_y^2 + a_z^2\right) / \left(b_x^2 + b_y^2 + b_z^2\right)$$

Using the coefficients in the Supplementary Figure 7, we arrive to I_{fund}/I_{ovtn} of 669, 937, 3839, and 847 for mode 86, 89, 92, and 93, respectively. These ratios are consistent with those calculated from Scheme C in Table S1. In view of the fact that the experimental overtone C-C bands from the retinal are only 50, 80 110 and 60 times smaller than for the fundamental bands, respectively, and assuming that all the experimental intensity of the overtone C-C bands originate from electrical anharmonicities, we overestimated 3-6 fold the ratio $\|\boldsymbol{a}\|/\|\boldsymbol{b}\|$ in our calculation, i.e., the weight of the second-order components (electrical anharmonicity) should be 3-6 times larger than calculated. Even if we consider than only around half of the experimental overtone intensity originates from electrical anharmonicities, the weight of the second-order components should be still 2-3 larger than calculated to explain the experimental results.

2 Supplementary Figures and Tables

2.1 Supplementary Figures

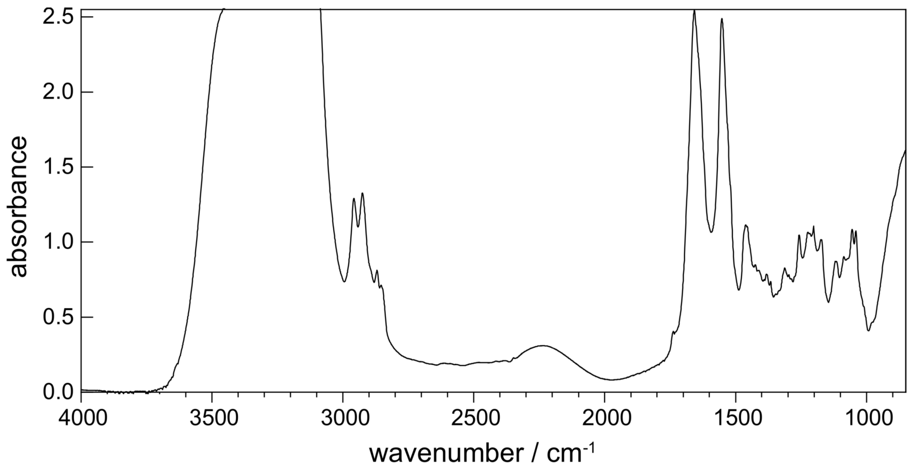

Supplementary Figure 1. Absorption FT-IR spectrum at 80 K of the hydrated film of BR in purple membranes used in the present study. The film was thicker than usually, leading to an absorbance above 2 in several spectral regions, preventing to obtain reliable difference absorption spectra in these regions. However, it also led to an increase of the signal from overtone and combination bands studied in this work.

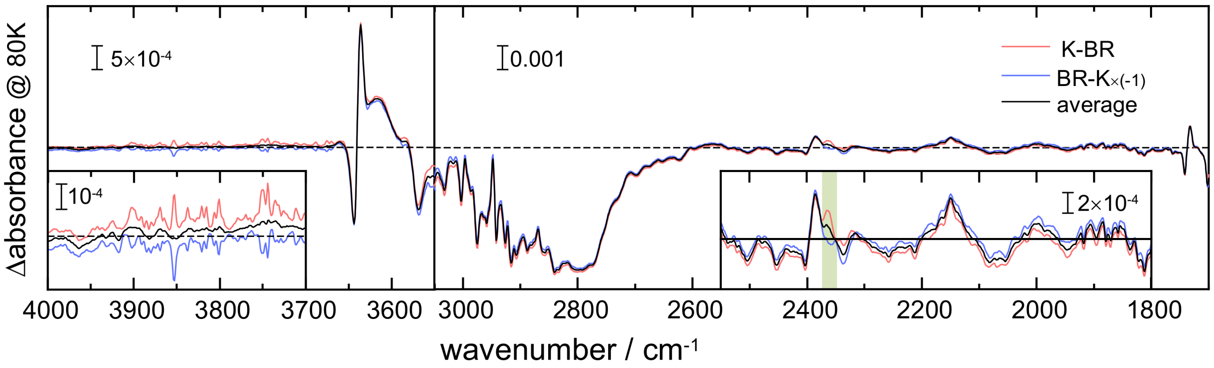

Supplementary Figure 2. K-BR (red trace), minus BR-K (blue trace), and the averaged K-BR difference FT-IR spectrum (black trace). Note that both the K-BR (red trace) and the minus BR-K (blue trace) difference spectrum have identical signals but opposed vapor and CO_2 contributions (see left insert, and the green area in the right insert, respectively), with their average canceling both contributions.

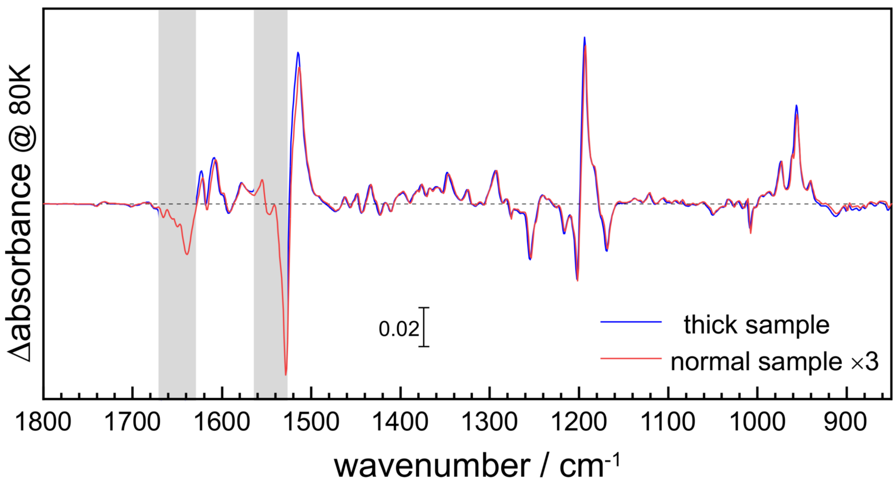

Supplementary Figure 3. Light-induced FT-IR difference K-BR spectrum at 80K, obtained using a normal (red trace) or a thick (blue trace) film sample. For the thick sample, the absorption changes in strongly absorbing regions were not reliable (indicated by gray vertical lines) and are omitted. However, the thicker sample provided three times larger signals and, thus, better signal-to-noise in some other regions, without any apparent spectral alterations.

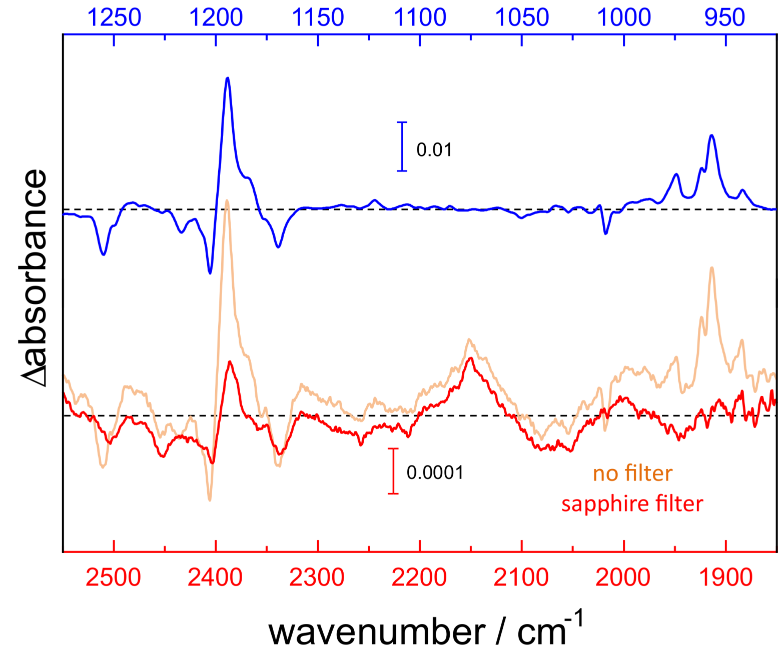

Supplementary Figure 4. Comparison of the 2550-1850 cm^{-1} (orange trace) and the 1275-925 cm^{-1} (blue trace) regions of a K-BR difference spectrum obtained with a sample of normal thickness in an old FT-IR spectrometer model (Biorad FTS-40) with an optical design not optimized to prevent back-reflections to reach the detector. The same spectrum was obtained placing a sapphire window (red trace), which removes any double-modulation artifact bands from around 3200 to 1800 cm^{-1}.

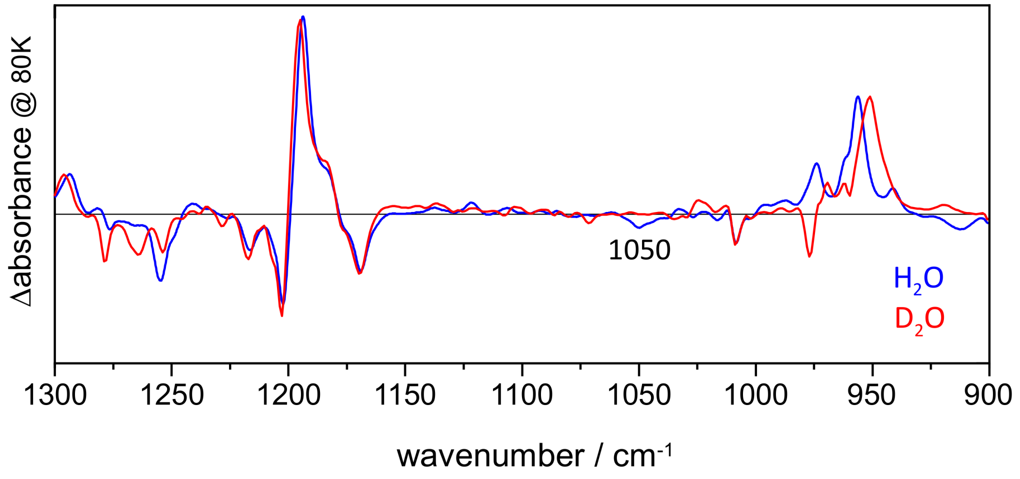

Supplementary Figure 5. K-BR difference FT-IR spectrum, obtained with a sample hydrated with H_2O (blue) and D_2O (red). Hydration with D_2O only deuterates the N-H group from the retinal. The disappearance of the negative band at 1050 cm^{-1} in D_2O indicates, when also considering its wavenumber, that this band originates from a vibration with contributions from an N-H bend.

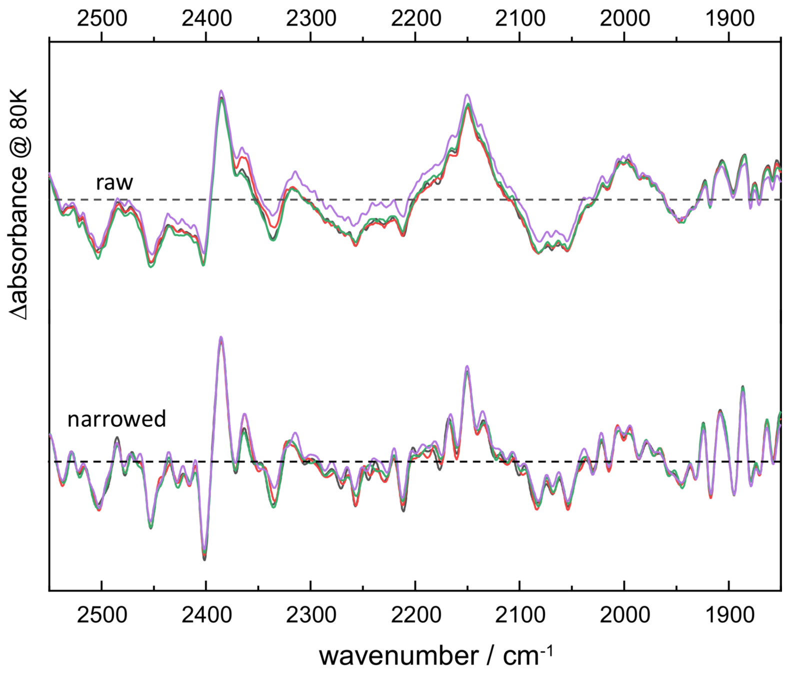

Supplementary Figure 6. Reproducibility of the K-BR difference FT-IR spectrum, both before and after band-narrowing. The figures displays four measurements, each of them the result of averaging K-BR and minus BR-K difference spectra.

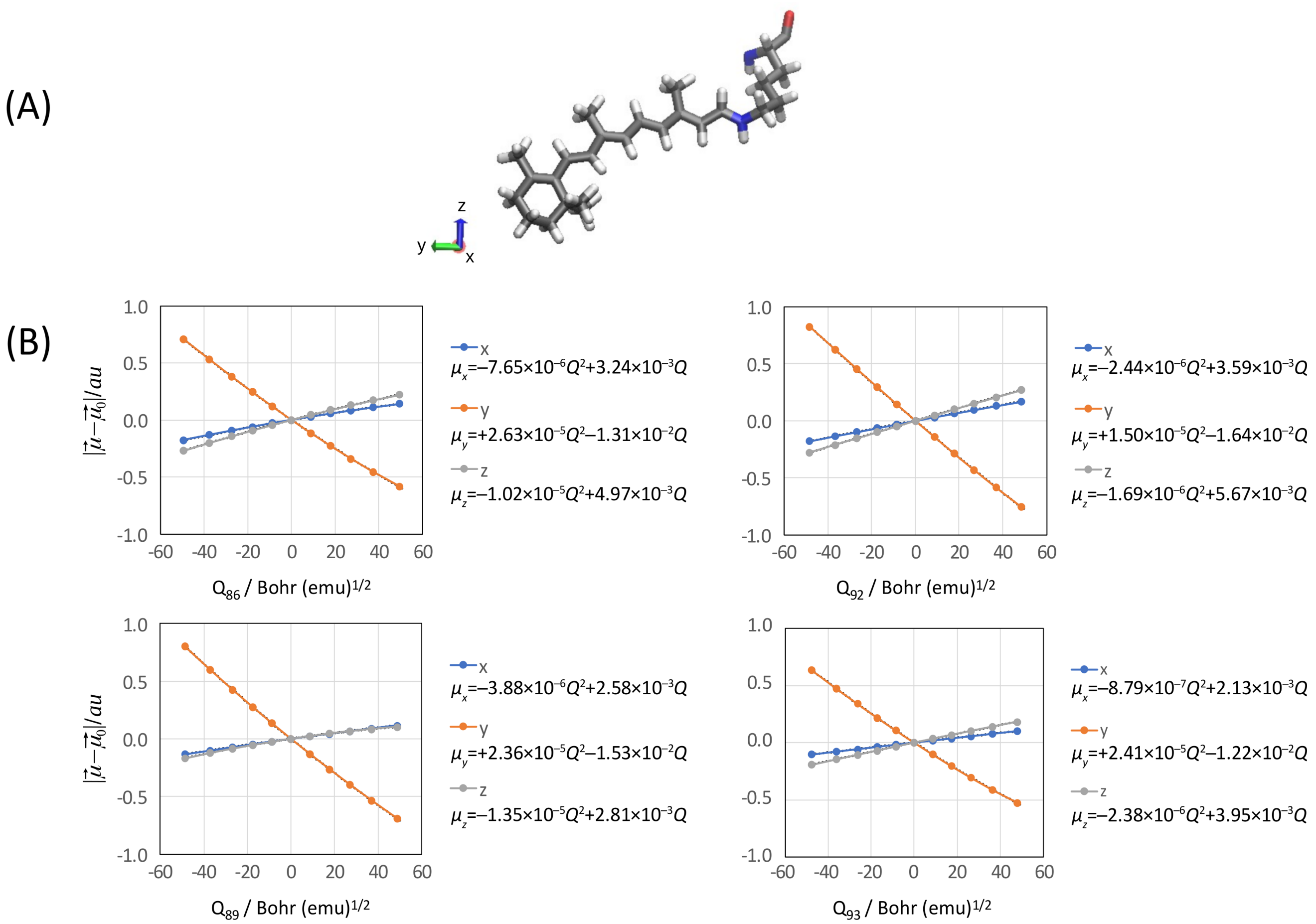

Supplementary Figure 7. Electrical anharmonicity for four retinal C-C stretching modes. (A) Orientation of the retinal to respect the selected Cartesian coordinate system, roughly aligning the *y* axis along the retinal C-C bonds. (B) Calculated variation of the *x*, *y*, and *z* components of the dipole moment as a function of displacement from the equilibrium geometry (circles with connected solid lines). The vibrational modes considered are: Q_{86} (10C-11C), Q_{89} (14C-15C), Q_{92} (8C-9C), and Q_{93} (12C-13C/14C-15C). The dependence of the dipole moment on a vibrational coordinate was fitted to a second-order polynomial, with first and second-order coefficients shown in the legends.

2.2 Supplementary Tables

Supplementary Table 1. Calculated frequencies and IR intensities of fundamental, overtone, and combination vibrational transitions of the retinal, using either scheme B (accounting for both mechanical and electrical anharmonicities) or scheme C (only accounting for electrical anharmonicities). For comparison, experimental values are included as well, following the assignments presented in Tables 1 and 2 from the main text.

Assignment	Exp[a]	State	Scheme B[a]		Scheme C		$\nu_C - \nu_B$ / cm^{-1}	I_C/I_B
	ν / cm^{-1}		ν_B / cm^{-1}	I_B / (km mol^{-1})	ν_C / cm^{-1}	I_C / (km mol^{-1})		
19C/20C rock	1008	72_1	1034	122.9	1034	174.3	–1	1.4
H15C=NH HOOP	1050	82_1	1108	63.6	1117	62.8	+9	1.0
10C-11C	1169	86_1	1211	344.9	1210	374.7	–1	1.1
14C-15C	1202	89_1	1252	261.2	1247	441.0	–5	1.7
8C-9C	1217	92_1	1264	771.3	1255	566.5	–8	0.7
12C-13C/14C-15C	1255	93_1	1300	297.1	1296	303.5	–3	1.0
19C/20C rock + 10C-11C	2175	72_1 86_1	2248	0.2	2244	0.3	–4	1.4
19C/20C rock + 14C-15C	2211	72_1 92_1	2302	1.3	2289	0.9	–13	0.7
19C/20C rock + 12C-13C/14-15C	2257	72_1 93_1	2337	0.2	2330	0.2	–7	1.0
$(10C\text{-}11C)_2$	2335	86_2	2422	1.4	2421	0.6	–1	0.4
14C-15C + 10C-11C	-	89_1 86_1	2466	0.5	2457	0.6	–9	1.2
8C-9C + 10C-11C	-	92_1 86_1	2478	1.2	2466	0.7	–12	0.6
$(14C\text{-}15C)_2$	2402	89_2	2503	0.8	2494	0.5	–10	0.6
8C-9C + 14C-15C	2416	92_1 89_1	2519	0.5	2502	0.4	–17	0.9
$(8C\text{-}9C)_2$	2427	92_2	2529	0.7	2511	0.1	–18	0.2
12C-13C/14C-15C + 14C-15C	2453	93_1 89_1	2556	0.5	2543	0.4	–13	0.8
12C-13C/14C-15C + 8C-9C	2402	93_1 92_1	2567	0.7	2552	0.5	–15	0.7
$(12C\text{-}13C/14C\text{-}15C)_2$	2503	93_2	2604	0.7	2593	0.3	–11	0.5

[a] Reproduced from Table 1 and Table 2 from the main text.

Supplementary Table 2. Contribution of VSCF (vibrational self-consistent field) configuration functions to calculated fundamental and overtone/combination excited states. In scheme B, fundamental and excited states had little mixing, with the main configuration being > 0.95 in its CI coefficient. In scheme A, in contrast, there is significant mixing with a second configurations for some fundamental and excited states.

Assigned State	Scheme B[a]			Scheme A[b]				
	ν_B / cm^{-1}	C_1[c]	Ψ_1[d]	ν_C / cm^{-1}	C_1	Ψ_1	C_2	Ψ_2
86_1	1211	+0.998	86_1	1199	+0.904	86_1		
89_1	1252	+0.983	89_1	1232	+0.784	89_1	–0.575	92_1
92_1	1264	+0.983	92_1	1225	+0.774	92_1	+0.545	89_1
93_1	1300	+0.983	93_1	1269	+0.950	93_1		
86_2	2422	+0.995	86_2	2396	+0.902	86_2		
89_1 86_1	2466	+0.983	89_1 86_1	2430	+0.651	89_1 86_1	–0.506	92_1 86_1
92_1 86_1	2478	+0.966	92_1 86_1	2423	+0.717	92_1 86_1	+0.513	89_1 86_1
89_2	2503	+0.991	89_2	2456	+0.606	89_2	–0.594	92_2
92_1 89_1	2519	+0.950	92_1 89_1	2462	+0.677	92_1 89_1	–0.563	89_2
92_2	2529	+0.957	92_2	2450	+0.598	92_2	+0.515	92_1 89_1
93_1 89_1	2556	+0.978	93_1 89_1	2498	+0.681	93_1 89_1	–0.631	93_1 92_1
93_1 92_1	2567	+0.979	93_1 92_1	2493	+0.682	93_1 92_1	+0.575	93_1 89_1
93_2	2604	+0.997	93_2	2535	+0.905	93_2		

[a] VCI@B3LYP-D3/cc-pVTZ (see Material and Methods in the main text).

[b] VQDPT2@B3LYP-D3/cc-pVDZ (see Material and Methods in the main text).

[c] CI coefficients

[d] VSCF configuration functions
